# Assessing undergraduate student and faculty views on animal research: What do they know, whom do they trust, and how much do they care?

**DOI:** 10.1371/journal.pone.0223375

**Published:** 2019-10-24

**Authors:** Eric P. Sandgren, Robert Streiffer, Jennifer Dykema, Nadia Assad, Jackson Moberg

**Affiliations:** 1 Pathobiololgical Sciences, School of Veterinary Medicine, University of Wisconsin-Madison, Madison, Wisconsin, United States of America; 2 Medical History and Bioethics, School of Medicine and Public Health, University of Wisconsin-Madison, Madison, Wisconsin, United States of America; 3 University of Wisconsin-Madison Survey Center, University of Wisconsin-Madison, Madison, Wisconsin, United States of America; Universidade do Porto Instituto de Biologia Molecular e Celular, PORTUGAL

## Abstract

Research using animals is controversial. To develop sound public outreach and policy about this issue, we need information about both the underlying science and people’s attitudes and knowledge. To identify attitudes toward this subject at the University of Wisconsin-Madison, we developed and administered a survey to undergraduate students and faculty. The survey asked respondents about the importance of, their confidence in their knowledge about, and who they trusted to provide information on animal research. Findings indicated attitudes varied by academic discipline, especially among faculty. Faculty in the biological sciences, particularly those who had participated in an animal research project, reported the issue to be most important, and they reported greater confidence in their knowledge about pro and con arguments. Among students, being female, a vegetarian/vegan, or participating in animal research were associated with higher ratings of importance. Confidence in knowledge about regulation and its adequacy was very low across all groups except biological science faculty. Both students and faculty identified university courses and spokespersons to be the most trusted sources of information about animal research. UW-Madison has a long history of openness about animal research, which correlates with the high level of trust by students and faculty. Nevertheless, confidence in knowledge about animal research and its regulation remains limited, and both students and faculty indicated their desire to receive more information from courses and spokespersons. Based on these findings, we argue that providing robust university-wide outreach and course-based content about animal research should be considered an organizational best practice, in particular for colleges and universities.

## Introduction

For pragmatic, epistemic, and democratic reasons, public policy about science should take into account the public’s views [1–3]. Sound policy design thus requires that policy makers be informed about the views of the public, and conversely, the public must have access to reliable and accurate scientific information from sources they listen to and trust. This latter requirement is especially important for scientific issues that are ethically controversial, as those will be viewed through a filter of people’s ethical and moral values rather than just based on facts [4–7]. Both goals can be met by encouraging robust and truthful dialog between policy makers and the public.

The use of animals in research is an ethically controversial scientific subject that may benefit from such dialog [8,9]. To this end, Hobson-West argues that use of public opinion in regulatory decision-making about animal research provides those decisions with rationale, moral legitimacy, and democratic legitimacy [10]. Germain and colleagues argue that openness may help to counter the perceived authoritarian character and “misalignment with public interest” of today’s science [11]. In Europe, two initiatives with over a hundred signatories have been established to track and promote openness by organizations that use animals in research [9,12]. Our organization, the University of Wisconsin-Madison (UW-Madison), has a 20+ year history of public discussion of animal research that is quite robust compared to most peer universities in the United States. This history includes debates between animal researchers and animal activists, the Forum on Animal Research Ethics (FARE), which over three and a half years invited 19 speakers with various perspectives to present publicly their views on the subject [13], in-house and traveling outreach programs sponsored by specific units on campus, and active engagement with the press to identify when animals have contributed to research breakthroughs but also to publicly acknowledge and explain incidents when animal research oversight has failed. Finally, UW-Madison supports a detailed website that presents general information about animal research results and regulations as well as specific controversies associated with animal research at the university (https://animalresearch.wisc.edu).

Establishing and maintaining effective public discussion requires that we respect the diverse demographic, ethical, and science-related underpinnings of people’s beliefs and attitudes about animal research. This approach is important for efforts to reach a societal consensus or, more realistically, to move closer to a respectful compromise about the circumstances under which animal research should be allowed. As members of a university community that conducts animal research and has a history of active public dialog about animal research controversies from multiple points of view, we developed a survey tool to acquire information that we could use to refine communication within the university community. We asked respondents to self-assess their knowledge of and views about animal research, whether they wanted additional information about animal research, and from whom they would trust any information provided [14].

With these considerations in mind, we designed our research to answer three general questions that should provide needed background information for the design of effective communication. (I) Do students and faculty care about animal research? (II) How much do they know about it? (III) From whom might they want to learn more? Specific questions were: (I) how important do students and faculty find the issue of animal research; (IIA) how confident are they in their knowledge about arguments for and against animal research; (IIB-IIF) how confident are they in their knowledge about how and how well animal research is regulated, and whether that regulation is adequate; (IIG) how confident are they that they possess the necessary information to make informed decisions about animal research; (IIIA) where do they get information about this topic; (IIIB) whom do they trust to be least biased; and (IIIC) do they want to know more?

Our study populations included two groups at the University of Wisconsin-Madison: a random sample of undergraduate students and a census of all faculty. We selected students as one target population because they represent the largest component of the campus community and are the primary focus of campus educational efforts. We selected faculty because they have dominant roles in setting campus policy related to research, as well as in selecting course content and setting the educational tone for discussions about controversial subjects.

Our findings point to a need and desire for additional outreach and educational initiatives, and can facilitate the design of strategies to improve the quality of campus-wide discussion about animal research. This, in turn, can guide the development of sound local and national policy. This is especially important in the United States, where transparency about animal research has been the exception rather than the rule [8]. More generally, our study answers the call for “new research in the humanities and social sciences to inform emerging discussions and priorities on the governance and practice of laboratory animal research, including on issues around… openness and public engagement…,” as called for in the recent publication “Developing a Collaborative Agenda for Humanities and Social Scientific Research on Laboratory Animal Science and Welfare,” authored by an interdisciplinary and international group [15]. Our report begins to address their research question 16, “Where are the opportunities for greater meaningful public and stakeholder engagement in the policy and practices of animal research?”, question 17, “What, and in what contexts, do different publics want to know about animal research?”, question 18, “How do people’s life experiences and other factors (e.g., profession, religion, pet-keeping) influence attitudes and behaviors around animal research?” and question 19, “What factors influence the construction of trust around animal research in diverse publics?” The audience for our report is far reaching, and will include animal care personnel, animal activists, scientists, ethicists, educators, spokespersons, administrators, and policy-makers in positions to influence how animal research is discussed, regulated, and, ultimately, practiced.

## Materials and methods

We conducted a web survey to gauge students’ and faculty’s attitudes and beliefs about animal research. Questionnaires were administered in English (only) and participation was voluntary. All aspects of this study were approved by the UW-Madison Education and Social/Behavioral Science Institutional Review Board.

### Student survey

For the 29,536 undergraduate students enrolled in the fall of 2016 at the University of Wisconsin-Madison, the University of Wisconsin Survey Center (UWSC) received a sample file containing non-FERPA protected information for each from the Bursar’s Office. Of these, 2,000 students were randomly selected from each year (freshman, sophomore, junior, and senior, measured by credits completed) to create a total sample of 8,000. Students were contacted via an email invitation sent by the UWSC with the subject line “UW-Madison Wants Your Thoughts on Animal Research!” The email was personalized with the student’s name. The study used an automatic login procedure for which the invitation included an embedded URL that was formatted to contain the student’s username and password [16], and that took the student directly to the on-line questionnaire. In addition to the email invitation, nonresponding students received three email reminders, each containing an embedded URL, spaced about every 10 days. The field period extended from September 29^th^ to November 22^nd^, 2016. In total, 782 students completed the questionnaire for an overall response rate of 9.8% [17].

### Faculty survey

A census of the 2,153 University of Wisconsin-Madison faculty was undertaken in the spring of 2017. Names and university postal and email addresses for university employees were obtained from the Data Resource Management Technology office at the university. University employees were selected for inclusion if they were identified as “Faculty” and were currently employed at the university. Faculty members were contacted up to five times. All faculty members were contacted initially with a postal letter printed on study-specific stationery that described the study’s purpose and included a URL and authentication credentials, which the faculty member could manually type into a browser to access the survey instrument. The letter also included a $2-bill cash incentive. Nonresponding faculty members received up to three emailed reminders to complete the questionnaire. Email procedures paralleled those described previously for the student sample. In a final attempt to secure participation, remaining faculty nonrespondents were sent a paper copy of the questionnaire in a postal mailing. In total, 942 faculty members completed the questionnaire for a response rate of 44%.

### Questionnaire

The existing literature contains numerous studies of public attitudes toward animals and of public attitudes toward animal research [18–30]. We reviewed several of the prominent surveys to compile a list of questions and coded them for approaches and themes. Sources included data from multiple years of Gallup Polls, Pew surveys, and Ipsos MORI surveys on public attitudes toward animal research and science in general. Based on this review of surveys, our questions then were developed with input from individual stakeholders within and outside of the university with backgrounds in animal research, animal research oversight, animal activism, bioethics, human-animal interactions, and survey design. We chose this diverse group to help ensure that question selection would not be unduly influenced by any one point of view on animal use in research. We selected questions that focused on the use of animals in research based on the purpose of the research (e.g., to test medications), the type of species (e.g., monkeys), and the extent of animal suffering. The final questionnaire included items about the prevalence of and trust in specific sources of information about the topic; past experience with being vegetarian or vegan and participating in animal research; the perceived importance of animal research; self-reported confidence in knowledge about animal research and its regulation; attitudes towards animals; the justifiability of specific research objectives and of using certain species; acceptable amounts of pain; the perceived adequacy of rules and regulations; information sources; and socio-demographic questions (e.g., gender and age).

The students’ and faculty’s questionnaires were identical except for two demographic items. First, to identify each individual’s academic discipline, students were asked for their current or anticipated major(s) (used to impute discipline), whereas faculty academic discipline was provided by the university. Second, students were asked for year in college, while faculty were asked for rank (assistant, associate, or full professor).

### Statistical analyses

Statistical analyses were conducted with STATA. Bivariate analyses used Wilcoxon/Mann-Whitney tests and Kruskal-Wallis tests. These analyses are the non-parametric equivalents to t-tests and one-way ANOVA tests, respectively. Unlike parametric tests, Kruskal-Wallis and Mann-Whitney tests do not make any assumptions about the dependent variables being normally distributed. Since most of the dependent variables are ordinal, we use an ordinal logistic regression model for our estimation with a proportional odds assumption of identical log-odds ratio across various categories of the dependent variable. The regression also assumes an ordinal dependent variable such as a seven- or five-point scale with response categories moving from a lower to higher rank or vice versa. For dichotomous dependent variables we use logistic regression for estimation. Significance is indicated with the following notation: * p < 0.05; ** p < 0.01; *** p < 0.001.

## Results

### Study participants and study questions

For students, self-reported majors were grouped into four broad disciplinary divisions: biological sciences, physical sciences, social sciences, and arts and humanities. Forty-four students with undeclared majors were not assigned to a discipline and were excluded from our analyses of discipline effects. Faculty division was provided by UW-Madison. Numbers (as total “n” or %) and categories of participants are listed in Table 1.

**Table 1:** Categories and numbers of survey participants^1^.

Participant category	Student, n %	Faculty, n %
**All, n**	782	942
**Demographics %**		
Gender		
Male	36.7	65.8
Female	61.6	31.1
Discipline		
Biological Science	44.5	38.4
Physical Science	20.2	19.6
Social Science	22.5	24.2
Arts and Humanities	7.2	17.8
Year in school		
Freshman	29.7	n/a
Sophomore	22.0	n/a
Junior	24.9	n/a
Senior	23.4	n/a
Faculty rank		
Assistant Professor	n/a	20.4
Associate Professor	n/a	19.4
Full Professor	n/a	60.2
**Animal experiences %**		
Dietary preferences		
Vegetarian/vegan, last 5 years	19.6	16.6
Not vegetarian/vegan	80.4	83.4
Animal research experience		
Done animal res. project	14.0	29.8
Not done an. res. project	86.0	70.2
**Support for animal research %**: “I do not think that there is anything wrong with using animals in medical research.”		
Agree	43.4	60.6
Neither agree nor disagree	21.7	18.6
Disagree	35.0	20.9

^1^Some respondents did not answer every survey question, so subcategories do not always add up to their population total.

The proportion of female student respondents was 62% compared to 52% in the undergraduate population. Faculty respondents were proportional to gender, discipline, and rank in the total population.

To address our first general research question, we asked respondents to rate the importance of animal research (question I, Table 2).

**Table 2:** Survey questions.

Ques.	Question wording	Response categories
I	Many kinds of research studies conducted at UW-Madison and other institutions use animals. How important to you is the issue of using animals in research?	1 = Not at all important; 2 = A little important; 3 = Somewhat important; 4 = Very important; 5 = Extremely important
IIA	How much do you feel you know about the facts and arguments for and against the use of animals in research?	1 = Nothing; 2 = A little; 3 = Some; 4 = Quite a bit; 5 = A great deal
IIB	How much do you feel you know about the rules and regulations regarding the use and welfare of animals in research at UW-Madison?	1 = Nothing; 2 = A little; 3 = Some; 4 = Quite a bit; 5 = A great deal
IIC	In your opinion, how well does UW-Madison enforce federal laws and guidelines about the use and welfare of animals in research?	1 = Not at all well; 2 = A little well; 3 = Somewhat well; 4 = Very well; 5 = Extremely well; -1 = Don’t know
IID	In your opinion, how thoroughly does UW-Madison review proposals for animal research in order to decide whether or not the research should be conducted?	1 = Not at all; 2 = A little; 3 = Somewhat; 4 = Very; 5 = Extremely; -1 = Don’t know
IIE	In your opinion, how often does UW-Madison take steps to minimize [physical or emotional pain or suffering/pain or distress] of animals used in research at UW-Madison?	1 = Never; 2 = Rarely; 3 = Sometimes; 4 = Usually; 5 = Always; -1 = Don’t know
IIF	In your opinion, are the rules and regulations regarding the use and welfare of animals in research at UW-Madison…	1 = excessive and should be reduced? 2 = adequate and should be maintained? 3 = not tough enough and should be strengthened? -1 = Don’t know
IIG	To what extent do you feel you have the information you need to make informed decisions about when, how, or if at all animal research can be acceptable?	1 = Not at all; 2 = A little; 3 = Somewhat; 4 = Quite a bit; 5 = A great deal; -1 = Don’t know
IIIA	IIIA. Have you ever seen, heard, or read anything about the use of animals in research from any of the following sources? [a] News media in newspapers, television, radio, or online [b] Advertisements such as those on billboards or buses [c] Social media websites such as Facebook or Twitter [d] Friends, family, peers, or co-workers [e] From UW-Madison courses [f] From any UW-Madison official spokespersons [g] From any animal activist groups such as the Alliance for Animals and the Environment, PETA (People for the Ethical Treatment of Animals), or HSUS (Humane Society of the US)	1 = Yes; 2 = No
IIIB	How much do you trust each of the following sources to provide unbiased information about the use of animals in research? (a-g sources as above)	1 = Not at all; 2 = A little; 3 = Some; 4 = Quite a bit; 5 = A great deal
IIIC	Do you feel the amount you have seen, heard, or read about the use of animals in research from each of the following sources has been too little, just enough, or too much? (a-g sources as above)	1 = Too little; 2 = Just enough; 3 = Too much
IVA	To what extent do you agree or disagree with the following statement about the use of animals? I do not think that there is anything wrong with using animals in medical research.	1 = Strongly agree; 2 = Agree; 3 = Neither agree nor disagree; 4 = Disagree; 5 = Strongly disagree
IVB	In the past 5 years, have you ever… [a] been a vegetarian or vegan? [b] worked on a research project that used animals?	1 = Yes; 2 = No

To address our second general research question about respondent knowledge, we asked eight specific questions. First, we asked faculty and students about their confidence in their knowledge about arguments for and against animal research (question IIA, Table 2). This and several subsequent questions do not measure actual knowledge, but rather self-perceptions of extent of knowledge. We selected this approach because perceived extent of knowledge likely will be a primary driver of perceived need for additional information about animal research, one of our later research questions. Next, we asked students and faculty a series of questions by which they could self-report their overall and specific knowledge in this area. A critical component of informed discussion about animal research is awareness of the current rules and regulations that govern its practice: gaps in perceived knowledge become relevant content for outreach. The first of these questions asked about confidence in general knowledge about this area (IIB). The next three specific questions asked for opinions on how well UW-Madison complied with laws and regulations (IIC-E). Answers to these questions can guide the content and delivery of outreach efforts on this topic and can prompt UW-Madison to respond in a constructive and timely manner to concerns within its own community about the protections and care it provides for research animals. The remaining question about rules and regulations asked about their perceived adequacy or sufficiency (IIF). Finally, we asked respondents whether they felt their overall knowledge could support good decisions about animal research (IIG).

Our third general research question asked about sources of information on animal research. Designing an appropriate communication strategy and engaging in effective public dialog require that we understand not just how much our audiences know, but also their sources of information on the issue, how much they trust those sources to provide unbiased information, and whether they are interested in hearing more from them. We asked students and faculty three specific questions designed to give us this information. First, we asked them to identify their sources of information (IIIA, Table 2). Second, we asked them about the extent of their trust in sources of information, a critical element in effective communication. (IIIB). A third consideration when designing outreach strategies is the extent of interest in additional communication within the target audiences (IIIC).

In addition to these specific research questions, we wanted to evaluate the effect of certain respondent characteristics on their survey answers. First, as part of an assessment of general attitudes toward use of animals by humans, we asked respondents to comment on a statement about their pre-existing support of animal research taken from Herzog and colleagues’ animals attitude scale [31] (IVA). Second, to identify respondents’ ratings of the importance of animal research as a function of their experiences with animals, we asked them to answer two questions about dietary preferences and animal research experience (IVB).

### Research questions

#### Question I: The importance of animal research

**Fig 1:**
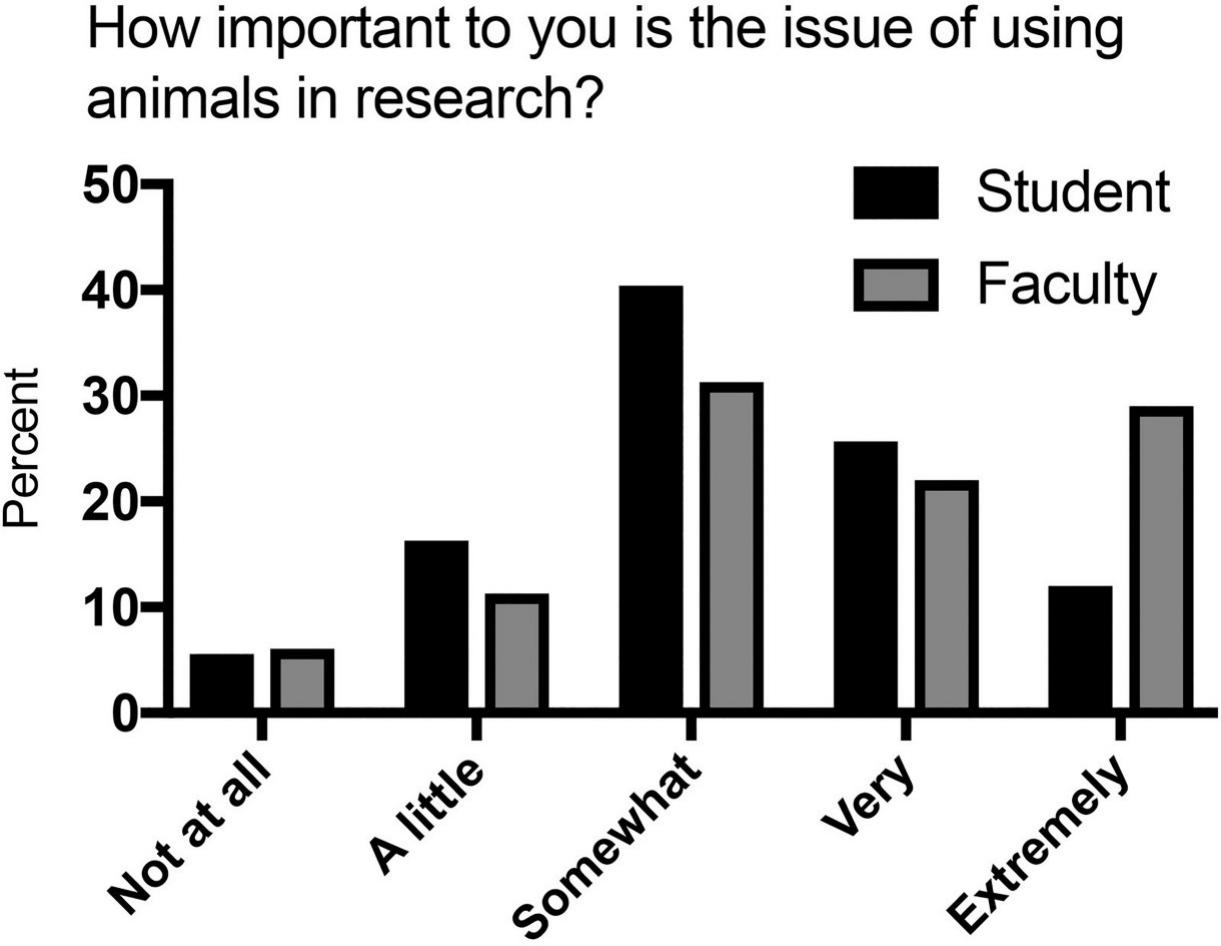
Importance of the issue of using animals in research.

The distributions of answers for students and faculty to our first general question “How important to you is the issue of using animals in research?” are shown in Fig 1. Thirty-seven percent of students and 51% of faculty identified the issue as “very important” or “extremely important”. Bivariate and multivariate analyses demonstrated that importance varied by gender only for students (Table 3; S1 Table). Variation by academic discipline was observed for both students and faculty, but this difference did not persist for students in the multivariate analysis. Faculty in the biological sciences found the issue much more important than any other discipline (odds ratios compared to biological science of 0.10 to 0.14). No differences were associated with student year in school or faculty rank (S1 Table).

**Table 3:** Importance of, knowledge about arguments for and against, and ability to make informed decisions about using animals in research.

Respondent characteristics	Importance X (SD), (1–5 scale)^1^		Arguments X (SD), (1–5 scale)^2^		Decisions X (SD), (1–5 scale)^3^	
	Students	Faculty	Students	Faculty	Students	Faculty
All	3.2 (1.0)	3.6 (1.2)	3.0 (1.0)	3.4 (1.0)	2.8 (1.2)	3.4 (1.2)
Male	2.9 (1.0)	3.6 (1.2)	2.9 (1.0)	3.4 (1.1)	3.0 (1.1)	3.5 (1.2)
Female	3.4 (1.0)	3.6 (1.1)	3.0 (0.9)	3.2 (1.0)	2.7 (1.2)	3.1 (1.2)
Odds Ratio vs. male	2.3***	0.76 ns	0.92 ns	0.66 ns	0.58*	0.57*
Biological Science	3.4 (1.0)	4.3 (1.0)	3.2 (1.0)	4.0 (0.9)	3.0 (1.2)	4.1 (1.0)
Physical Science	3.0 (1.0)	3.1 (1.2)	2.8 (0.9)	2.9 (1.0)	2.7 (1.1)	3.0 (1.2)
Odds Ratio vs. Bio	0.82 ns	0.10***	0.41***	0.09***	0.47**	0.12***
Social Science	3.2 (1.0)	3.1 (1.1)	2.8 (0.9)	3.0 (0.9)	2.5 (1.2)	2.9 (1.2)
Odds Ratio vs. Bio	0.85 ns	0.11***	0.27***	0.14***	0.37**	0.14***
Arts and Humanities	3.3 (1.0)	3.2 (1.0)	2.9 (0.9)	2.9 (0.8)	2.7 (1.2)	2.7 (1.0)
Odds Ratio vs. Bio	1.1 ns	0.14***	0.84 ns	0.12***	0.77 ns	0.10***
I do not think that there is anything wrong with using animals in medical research.^4^						
Agree	3.0 (1.1)	3.7 (1.2)	3.2 (1.0)	3.6 (1.0)	3.1 (1.2)	3.7 (1.2)
Odds Ratio vs. N	1.2 ns	2.1***	2.9***	2.4***	3.0***	2.9***
Neither (N)	2.9 (0.8)	3.1 (1.0)	2.6 (0.8)	2.9 (0.9)	2.4 (1.0)	2.8 (1.2)
Disagree	3.7 (0.9)	3.6 (1.0)	3.0 (0.9)	3.2 (1.0)	2.7 (1.1)	2.9 (1.2)
Odds Ratio vs. N	4.3***	2.4***	2.5***	1.9***	1.7*	1.5*

ns, not significant

* p<0.05

** p<0.01

*** p<0.001. From S1, S2 and S8 Tables.

^1^Higher numbers indicate greater self-reported importance.

^2^Higher numbers indicate greater self-reported knowledge of arguments for and against.

^3^Higher numbers indicate greater self-reported knowledge to make informed decisions; excludes those answering “Don’t know”, which was 12% of students and 9% of faculty.

^4^Agree = strongly agree and agree; N = neither agree nor disagree; Disagree = strongly disagree or disagree.

Taking the answer to the question “I do not think that there is anything wrong with using animals in medical research” (question IVA) as a general indicator of extent of support for animal research by respondents, we examined the corresponding distribution of answers about the importance of animal research as a function of that support. To simplify this assessment, we grouped agree and strongly agree into an “agree” category. Similarly, the “disagree” category was composed of disagree and strongly disagree. As shown in Table 3 for faculty, importance was highest among both supporters and non-supporters compared to those who neither agreed nor disagreed. Students who did not support animal research indicated a higher level of importance for this issue.

**Fig 2:**
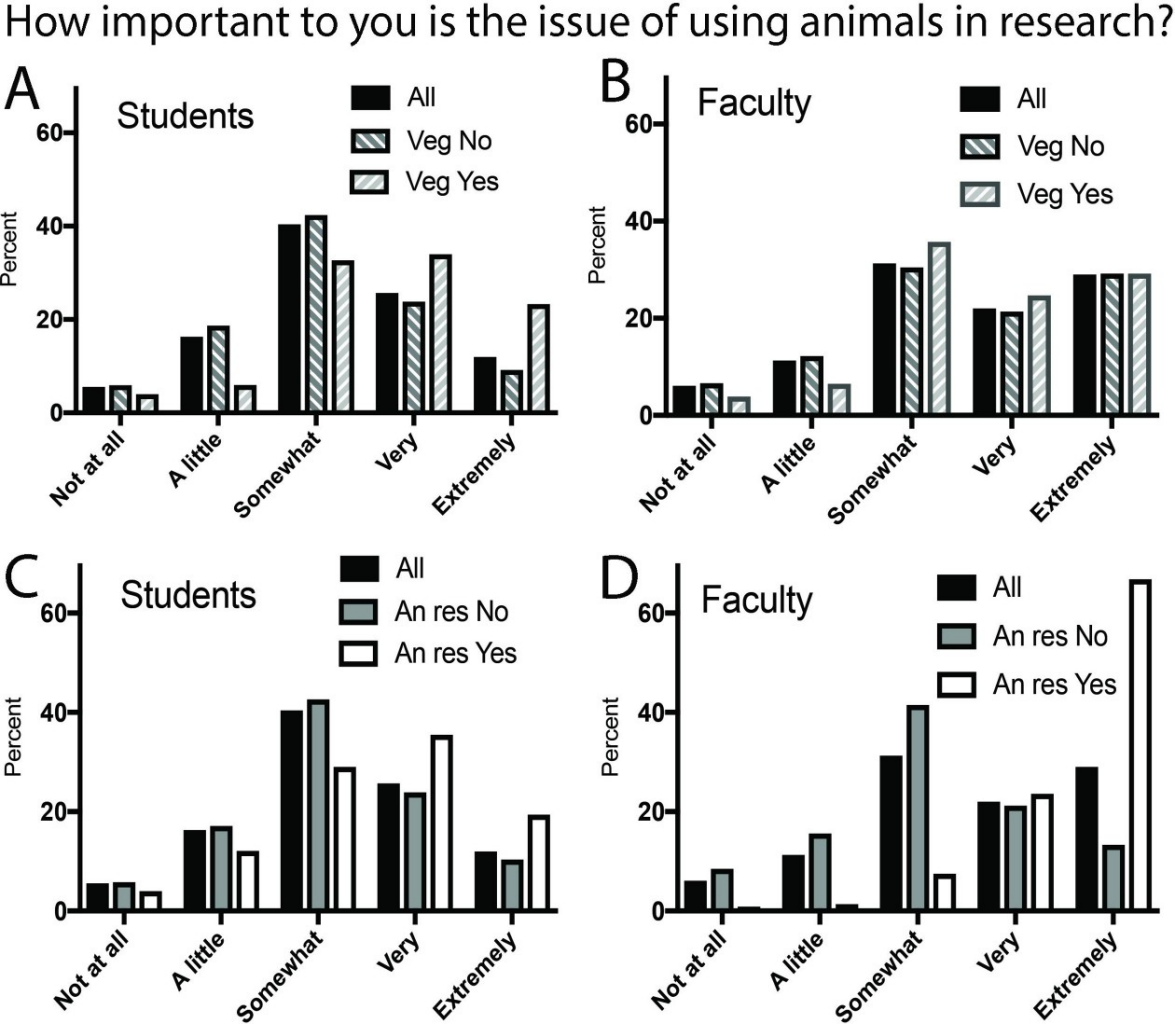
Dietary and animal research experience influences on importance of animal research. (A) Students and diet. (B) Faculty and diet. (C) Students and animal research experience. (D) Faculty and animal research experience.

Fig 2A–2D illustrate the distribution of importance scores for students and faculty based on their answers to questions about experiences with animals. Students who answered yes when asked if they were vegetarians/vegans or if they had done animal research assigned much higher levels of importance compared to those who answered no. For faculty, dietary choice had almost no effect on level of importance. Having worked on an animal research project was associated with a dramatic shift toward greater importance, with two-thirds of faculty respondents in this category indicating that animal research was an extremely important issue.

After assessing how much respondents cared about the issue of animal research, we asked a series of specific questions to address our second general question about how much respondents perceived they know about critical aspects of the issue.

#### Question IIA: Knowledge about arguments for and against animal research

**Fig 3:**
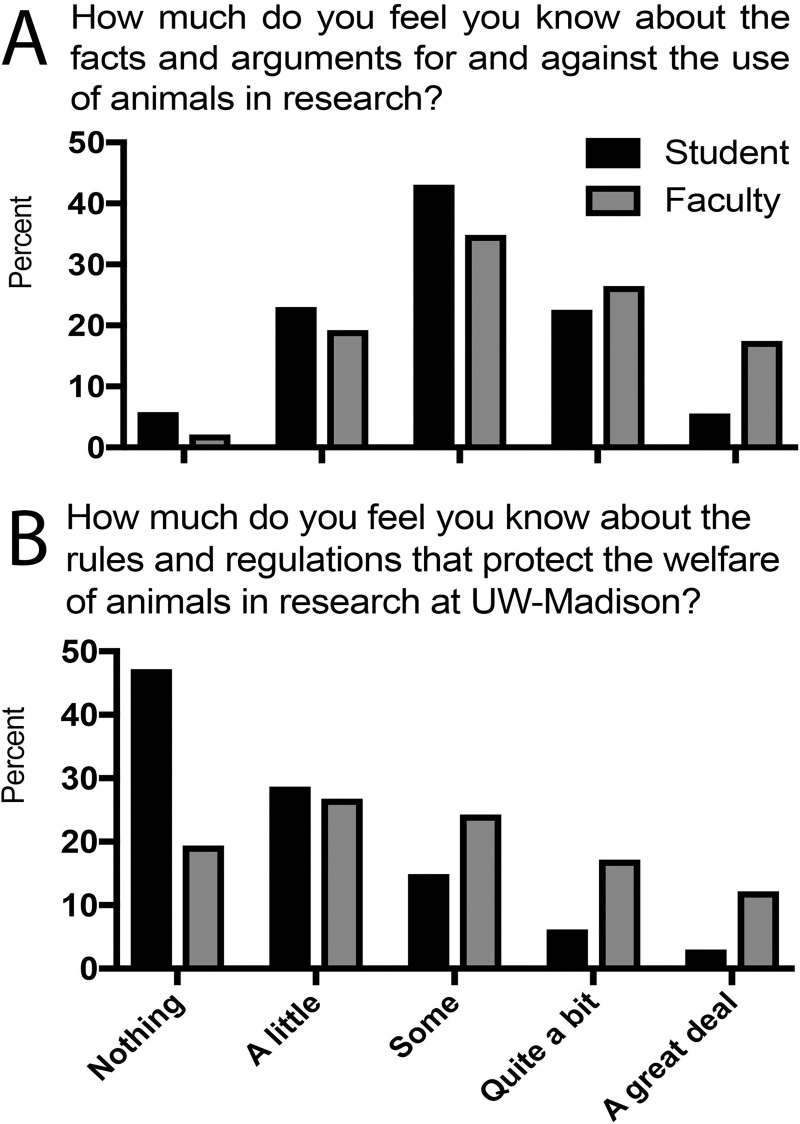
Knowledge about animal research. (A) Knowledge about facts and arguments for and against and about rules and regulations governing animal research. (B) Knowledge about rules and regulations that protect the welfare of animals in research.

For students, responses to the question “How much do you feel you know about the facts and arguments for and against the use of animals in research?” had a mean near the middle of the scale at “some” (Fig 3A). Faculty expressed greater self-reported knowledge about arguments than did students. For this question, there were no gender differences among students or faculty (Table 3; S2 Table). Self-reported knowledge was higher among both students and faculty in the biological sciences compared to other disciplines except arts and humanities for students. Self-reported knowledge of arguments significantly increased from freshman to juniors or seniors, and slightly with higher faculty rank (S2 Table). Self-reported knowledge about arguments was highest among both supporters and opponents of animal research as compared to those in the middle.

Although just under 10% of students who received the survey responded, rankings of the extent of importance of animal research and the extent of knowledge about arguments for and against animal research were normally distributed with the mean near the scale midpoint for those who did respond. These findings indicate that student respondents to the survey represent the full range of positions on overall importance and knowledge of pros and cons of this issue.

#### Question IIB: Knowledge about rules and regulations

**Fig 4:**
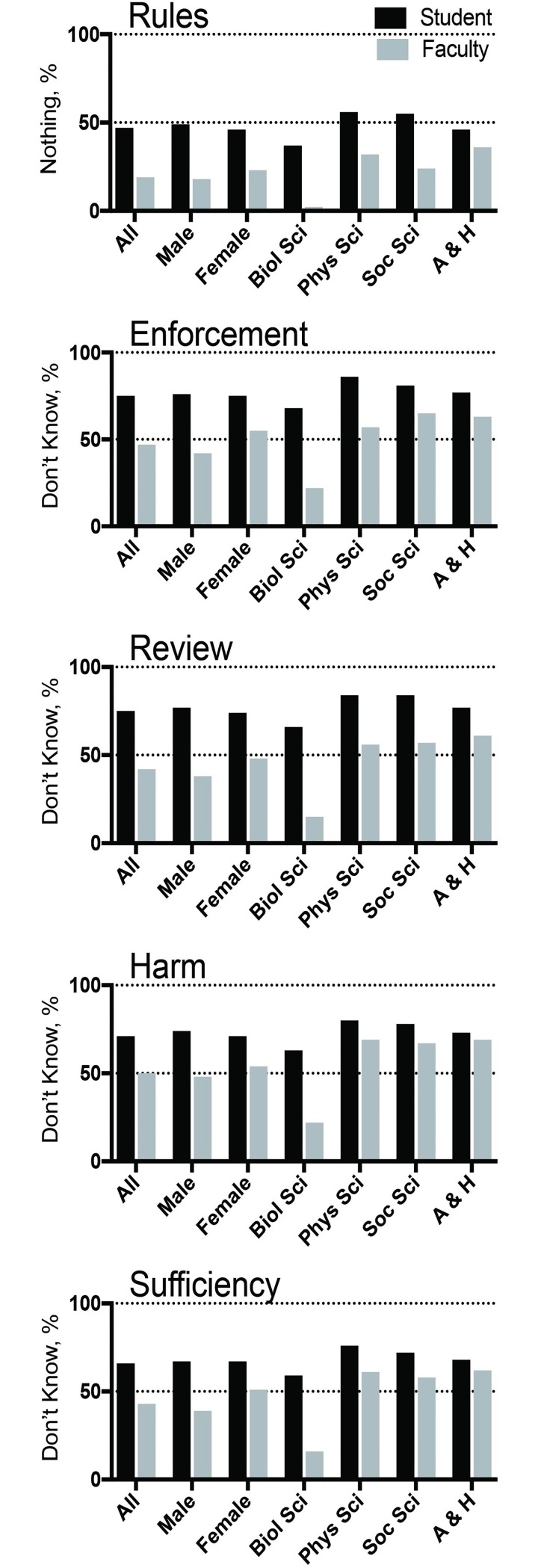
Percent of respondents answering “Nothing” about knowledge of rules and regulations, and “Don’t know” about adequacy of enforcement of laws, review of proposals, minimizing harm, and sufficiency of rules.

For the question “How much do you feel you know about the rules and regulations regarding the use and welfare of animals in research at UW-Madison?”, most striking for students was the large fraction (47%) who answered “nothing” to this question (Figs 3B and 4; S3 Table). This answer was not significantly different between genders, but decreased as students progressed from sophomores to seniors. The greatest variation was among academic disciplines, and odds for answering “nothing” for all disciplines were 2.4- to 3.2-times higher than for biological sciences. Among students expressing an opinion, average scores were below “some” (score of 3) (Table 4). Scores in biological sciences were higher than in all other disciplines, but the difference was significant only for physical sciences. For faculty, many trends were similar (Figs 3 and 4; Table 4; S3 Table). Just under one-fifth of all faculty reported they knew “nothing” about the rules and regulations. Compared to biological sciences, faculty in other disciplines had 14- to 29-times higher odds for answering “nothing” to this question. Assistant professors gave this answer more often than associate or full professors. Among faculty expressing an opinion, the mean score was just above “some”. As for students, faculty in the biological sciences indicated the greatest extent of knowledge. Finally, students who disagreed and faculty who agreed or disagreed with animal research indicated greater knowledge (Table 4; S3 Table).

**Table 4:** Among those expressing an opinion, distribution of ratings of knowledge of rules and regulations, and adequacy of enforcement of laws, review of proposals, minimizing harm, and sufficiency of rules (all exclude respondents answering “Nothing” for knowledge of rules, or “Don’t know” for other questions).

Respondent character-istics	Rules X(SD) (2–5 scale)^1^		Enforcement X(SD) (1–5 scale)^2^		Review X(SD) (1–5 scale)^2^		Harm X(SD) (1–5 scale)^2^		Sufficiency X(SD) (1–3 scale)^3^	
	Stu	Fac	Stu	Fac	Stu	Fac	Stu	Fac	Stu	Fac
All	2.7 (0.9)	3.1 (1.1)	3.7 (1.0)	4.3 (0.7)	3.9 (1.0)	4.4 (0.7)	3.8 (1.1)	4.4 (0.8)	2.2 (0.6)	2.0 (0.5)
Male	2.7 (0.9)	3.3 (1.1)	3.9 (0.9)	4.3 (0.7)	3.9 (0.9)	4.4 (0.7)	4.1 (1.0)	4.4 (0.7)	2.0 (0.6)	1.9 (0.4)
Female	2.7 (0.9)	3.0 (1.2)	3.7 (1.0)	4.2 (0.8)	3.9 (0.9)	4.4 (0.6)	3.8 (1.0)	4.3 (0.8)	2.2 (0.6)	2.1 (0.5)
OR^4^ vs M	1.1 ns	0.67 ns	0.57 ns	0.59 ns	1.1 ns	0.83 ns	0.53 ns	1.0 ns	2.3*	1.4 ns
Bio. Sci.	2.9 (1.0)	3.8 (1.0)	4.0 (0.8)	4.5 (0.6)	4.1 (0.8)	4.5 (0.6)	4.0 (0.9)	4.6 (0.6)	2.2 (0.5)	1.8 (0.4)
Phys. Sci.	2.4 (0.8)	2.8 (0.9)	3.7 (0.7)	4.2 (0.6)	3.8 (0.9)	4.2 (0.8)	4.0 (0.9)	4.3 (0.9)	2.0 (0.7)	2.0 (0.4)
OR^4^ vs Bio	0.42*	0.12***	0.26*	0.36***	1.0 ns	0.35***	0.58 ns	0.57 ns	0.55 ns	3.2**
Soc. Sci.	2.4 (0.6)	2.7 (0.8)	3.3 (1.2)	4.1 (0.9)	3.5 (1.0)	4.4 (0.6)	3.6 (1.3)	4.1 (0.8)	2.2 (0.8)	2.2 (0.4)
OR^4^ vs Bio	0.39 ns	0.14***	0.34 ns	0.36**	0.52 ns	0.61 ns	0.85 ns	0.33***	1.6 ns	8.0***
A and H	2.6 (1.0)	2.6 (0.7)	2.8 (1.3)	3.7 (0.9)	3.1 (1.4)	4.0 (7.3)	3.1 (1.5)	3.8 (0.8)	2.2 (0.7)	2.4 (0.5)
OR^4^ vs Bio	0.74 ns	0.11***	0.17 ns	0.09***	0.9 ns	0.19***	0.13*	0.11***	0.46 ns	19***
I do not think that there is anything wrong with using animals in medical research.^5^										
Agree	2.9 (1.0)	3.4 (1.1)	4.1 (0.8)	4.4 (0.6)	4.2 (0.8)	4.5 (0.5)	4.2 (0.8)	4.5 (0.6)	2.0 (0.4)	1.9 (0.4)
OR^4^ vs N	2.6***	3.0***	1.5 ns	2.6***	2.5*	2.8***	2.1 ns	2.8***	0.60 ns	0.29**
Neither (N)	2.4 (0.6)	2.7 (0.8)	3.6 (1.0)	4.0 (0.7)	3.6 (1.0)	4.2 (0.7)	3.6 (1.1)	4.2 (0.7)	2.1 (0.4)	2.1 (0.5)
Disagree	2.5 (0.7)	2.9 (0.9)	3.1 (1.0)	3.8 (0.9)	3.3 (1.1)	4.1 (0.8)	3.3 (1.2)	3.7 (1.0)	2.5 (0.7)	2.4 (0.6)
OR^4^ vs N	1.1 ns	1.8 *	0.25**	0.74 ns	0.67 ns	0.69 ns	0.47 ns	0.48*	4.9***	4.1***

ns, not significant

* p<0.05

** p<0.01

*** p<0.001. From S2–S7 Tables.

^1^Higher numbers indicate greater self-reported knowledge

^2^Higher numbers indicate greater self-reported belief in quality of compliance

^3^Higher numbers indicate greater self-reported belief that regulations should be strengthened

^4^OR, Odds ratio using multivariate analysis

^5^Agree = strongly agree and agree; N = neither agree nor disagree; Disagree = strongly disagree or disagree.

#### Questions IIC-F Adequacy of compliance and regulations

Regarding adequacy of compliance (enforcement of laws, proposal review, minimization of harm to animals), most students and many faculty responded with “don’t know” for each of these questions (Fig 4; Table 4; S4 through S6 Tables). Faculty in the biological sciences were most likely to indicate some knowledge. Higher appointment status for faculty was most often positively correlated with the extent of knowledge, and student seniors and sometimes juniors indicated less uncertainty than freshmen. For all questions, students answered “don’t know” more often than faculty. Of those expressing an opinion about compliance, the mean value was above the midpoint (or 3.0) on each scale (Table 4; S4 through S6 Tables), with faculty in the biological sciences typically rating UW’s compliance with laws and guidelines more highly than those in other disciplines. Faculty expressed more confidence in compliance than students. Compliance scores were highest among faculty supporters of animal research.

As with the preceding questions, many respondents answered “Don’t know” to the question “In your opinion, are the rules and regulations regarding the use and welfare of animals in research at UW-Madison…1 = excessive and should be reduced; 2 = adequate and should be maintained; 3 = not tough enough and should be strengthened; -1 = Don’t know” (Fig 4; Table 4; S7 Table). Of remaining respondents, answers clustered around “adequate and should be maintained” (score of 2) (Table 4; S7 Table), with students expressing slightly less confidence (higher score) in the sufficiency of regulations than faculty. This result was based primarily on student-faculty differences in biological sciences, though in arts and humanities student confidence was actually higher than for faculty. For students, women were slightly less confident than men. Strong discipline differences were present for faculty, with biological science faculty most confident (lower score). Unsurprisingly, supporters of animal research were more confident and opponents less confident in regulatory sufficiency.

#### Question IIG: Adequacy of information to make informed decisions

**Fig 5:**
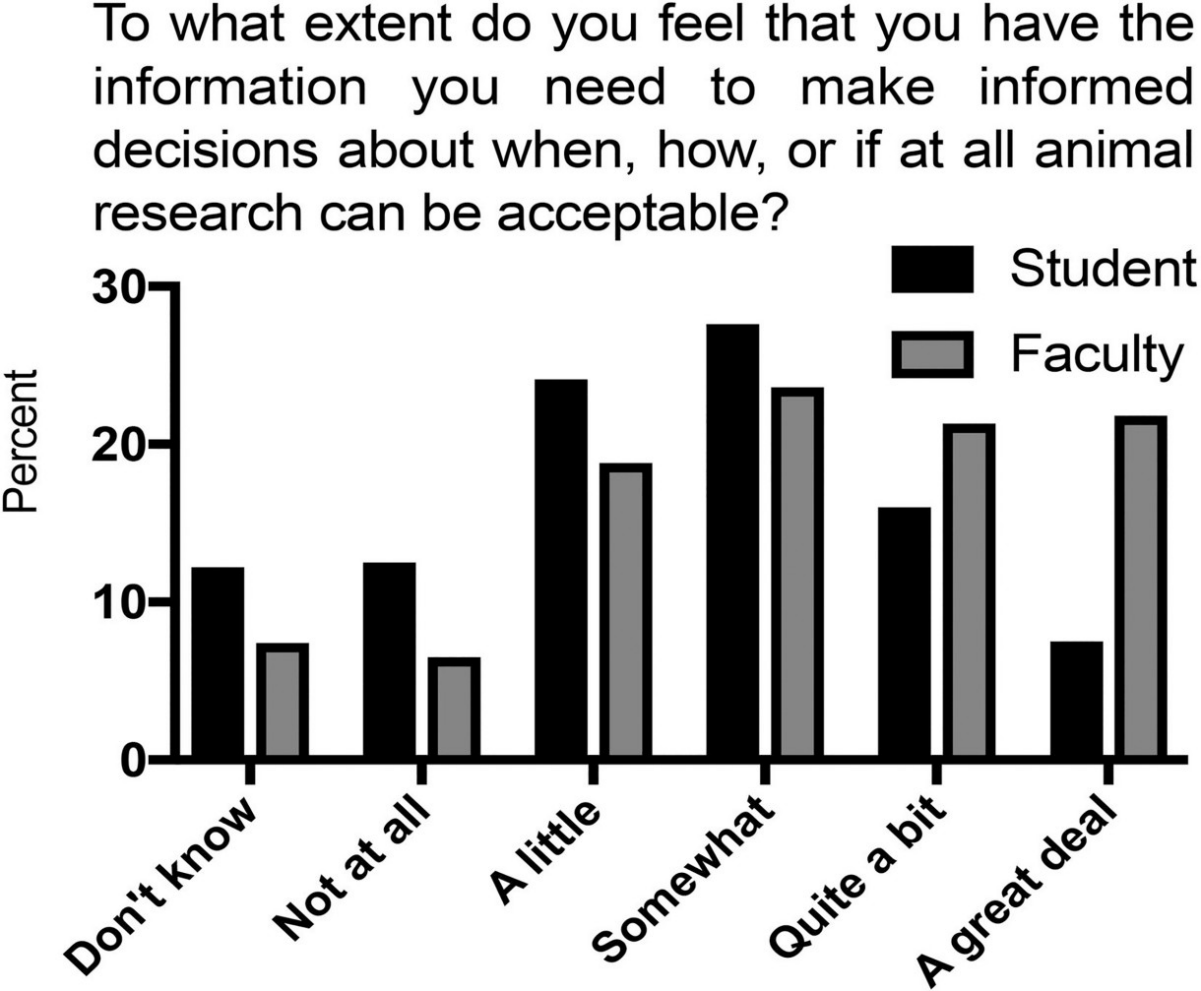
Possession of information necessary to make informed decisions about animal research.

To complete our assessment of general research question two, we asked respondents to evaluate the connection between their perceived knowledge and decision-making ability. Responses to the question “To what extent do you feel you have the information you need to make informed decisions about when, how, or if at all animal research can be acceptable?” are presented in Fig 5, Table 3, and S8 Table. A small percentage of respondents answered that they “did not know”(S8 Table). Compared to biological sciences, odds that faculty in other disciplines would answer the question with “don’t know” were 6- to 8-fold higher. Among those with an opinion, students’ self-identified knowledge sufficiency was normally distributed with a mean close to “somewhat” (score of 3), just below the center of the scale (Fig 5). For faculty, the curve was shifted to the right, with similar percentages answering “a little”, “somewhat”, “quite a bit”, and “a great deal.” In both groups, males expressed slightly more confidence in their knowledge than females, and biological sciences faculty and students expressed greater confidence in knowledge than those in other disciplines except for students in arts and humanities (Table 3). Overall, faculty were less likely to respond “don’t know” than students. Faculty with an opinion, especially in biological sciences, were more confident in their ability to make decisions than students except, again, in arts and humanities. Finally, agreement and disagreement with animal research acceptability both were correlated with increased confidence for both respondent populations.

After assessing respondents’ perceived knowledge of animal research, we asked questions to address our third general research question about sources of information on the topic.

#### Question IIIA: Sources of information

**Fig 6:**
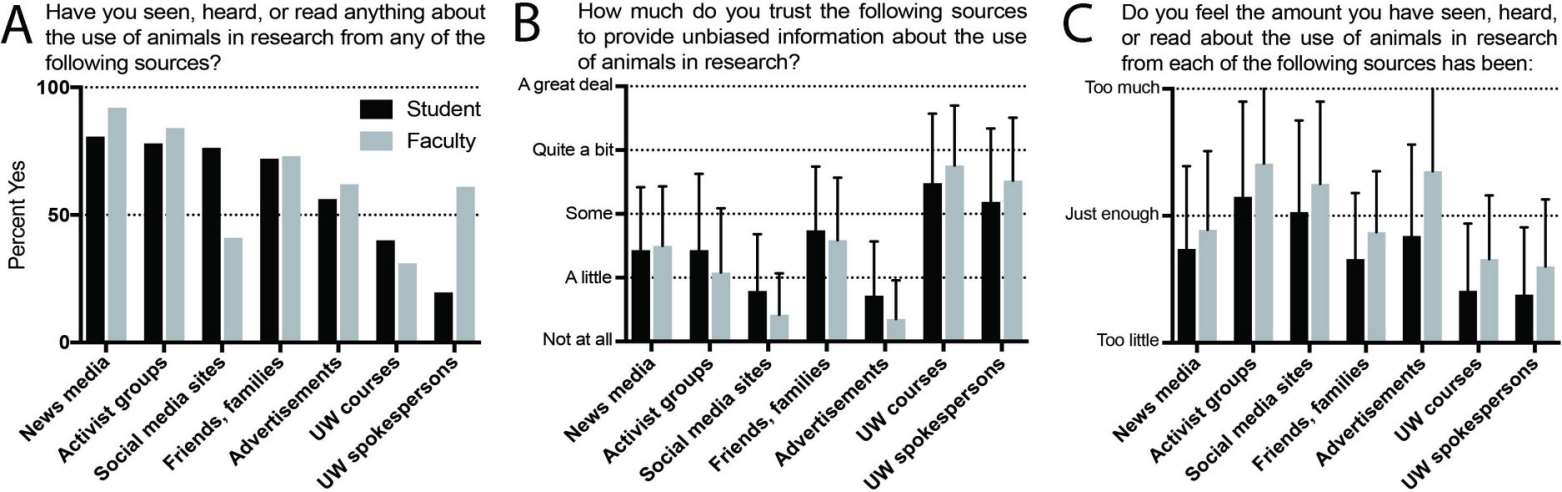
Sources of information. (A) Prevalence of sources of information about animals in research. (B) Trust in sources to provide unbiased information. (C) Sufficiency of information from each source. For B and C, data is shown as mean ± standard deviation.

Respondents were asked “Have you ever seen, heard, or read anything about the use of animals in research from any of the following sources?” Fig 6A displays common sources of information about animals in research in decreasing order of frequency for students. UW-Madison spokespersons are more commonly listed as a source for faculty than for students, likely related in part to faculty’s longer average time in residence on campus.

#### Question IIIB: Trust in sources

Strikingly, when asked “How much do you trust each of the following sources to provide unbiased information about the use of animals in research?”, both students and faculty answered that UW-Madison courses and UW-Madison official spokespersons were the most highly trusted sources (Fig 6B).

We also assessed trust in university spokespersons and in animal activist groups as a function of respondent support for animal research. There was an inverse relationship in source trust depending on level of support: supporters had greater trust in university spokespersons and less trust in animal activist groups, while the converse was true for opponents (Table 5). Interestingly, even among opponents of animal research, trust in university spokespersons was as high as or higher than trust in activist groups.

**Table 5:** Trust in sources of information about animal research as a function of respondent support for animal research.

	I do not think that there is anything wrong with using animals in medical research.							
Information source	Students (1–5 scale)				Faculty (1–5 scale)			
	Agree^1^ X (SD)	Neither^2^ X (SD)	Disagree^3^ X (SD)	P	Agree^1^ X (SD)	Neither^2^ X (SD)	Disagree^3^ X (SD)	P
Trust in UW-Madison spokespersons	3.5 (1.2)	3.3 (1.1)	3.0 (1.1)	27 ***	3.8 (0.9)	3.3 (1.0)	3.1 (1.1)	78 ***
Trust in animal activist groups	2.1 (1.1)	2.5 (1.1)	3.0 (1.2)	88 ***	1.8 (0.9)	2.3 (0.9)	2.7 (1.0)	135 ***

***, p<0.001 using Chi^2^

^1^Agree or strongly agree

^2^Neither agree nor disagree

^3^Disagree or strongly disagree

#### Question IIIC: Amount of information received

Respondents were asked “Do you feel the amount you have seen, heard, or read about the use of animals in research from each of the following sources has been too little, just enough, or too much?” They identified UW-Madison courses and official spokespersons as most likely to provide too little information out of all listed sources (Fig 6C). Animal activist groups and social media sites were seen by students and faculty as providing too much information, as were advertisements for faculty. From all sources, students expressed more desire for additional information than did faculty.

## Discussion

### Research questions

Effective communication about the animal research controversy requires identifying what information people need but lack to guide their decisions about when, if at all, animal research can be acceptable. Once communicators acquire this information, they need to prepare appropriate messages for their target audience using sources those audiences follow and trust, and from which they are interested in learning more [5,14]. This must be done in a manner that respects the varied ethical beliefs about animals, including humans, among members of the audience [4–7]. In this study, we describe our attempts to gather some of this information by developing a survey tool and administering it to a sample of university undergraduate students and all members of the faculty. The survey was designed to address three broad research questions about whether they think this issue is important, how confident they are in their knowledge about it, and who they trust to provide more information. Answers to these questions can help us to identify knowledge gaps and design appropriate and welcome communication. Our findings are relevant to campus-wide outreach efforts designed to improve transparency, public discussion, and science policy about animal research, particularly in the United States, where communication has been less transparent than in Europe [8].

First, we note several study limitations that are relevant to study interpretation. We surveyed undergraduate students and faculty at only one university, and did not include members of the general public. Thus, compared to the general public, our respondents had a higher average level of education. Higher education has been associated with greater support for animal research [32–34]. However, our university is comparable to many other doctoral universities at which a large fraction of all animal research is conducted, so our findings should be generalizable to other members of this group. Given the role that such universities have in supporting and practicing animal research and their influential position regarding policy at the local, state, and national levels, it is helpful to understand these communities even though they are not representative of the general public in all ways.

Next, student responses may have been self-selected for perceived importance of the issue. In particular, women represent 52% of the UW-Madison undergraduate student population, yet 62% of respondents were women, and female students rated the issue to be more important than men. (For faculty, the population and respondent genders were equivalent). Because of this gender discrepancy, together with the low response rate for students, we cannot assume that the student respondents’ answers to the questions are fully representative of the larger population. However, the student “n” of 782 is very high compared to most student surveys in this area. There is growing empirical evidence that high response rates, while undoubtedly important, are often not as important an indicator of reliability as once believed, provided the sample size is at least 500 individuals. For example, according to Fosnacht and colleagues [35], “[w]ith few exceptions, we found estimates for several measures of college student engagement to be reliable under low response rate conditions (5%-10%), provided the sampling frame included at least 500 students”, and “few major differences were observed in medium [500–1,000] or large [>1,000] administrations at low simulated response rates….” Also, for both importance and knowledge about arguments for and against the use of animals in research, the student responses were normally distributed with means near the middle of each scale, so our survey does capture the full range of student perspectives on each question.

As a final limitation, we note that much of the current debate about the justifiability of animal research arises from recent reports that biomedical research, including that using animals, often lacks scientific rigor, reproducibility, and translatability to human medicine [36–38]. Because our paper focuses on broader categories of information relevant to improving efforts for outreach and public dialog (which may well *implicitly* reflect respondents’ beliefs about translatability and reliability) rather than the specific content of those efforts, that question is not included in the present analysis. But we want to emphasize that people’s understanding of the arguments and counterarguments surrounding this issue, and the ways that understanding figures into people’s broader views about the ethics of animal research, are important areas for future research. We also note that, even if biomedical science could eliminate these faults (and thereby strengthen the benefit category of the typically utilitarian ethical assessment), animal research would remain ethically contentious and outreach and public dialog would be no less important.

Regarding our first research question about importance of this issue, we found that both students and faculty identified animal research as an important topic. Only 23% of students and 21% of faculty indicated the issue was “not at all important” or only “a little important”. Lund and colleagues [30] reported that, among focus groups in the Danish public, animal research was not very “salient”, or often on their minds. However, UW-Madison publicly discusses this issue frequently and has been repeatedly criticized by animal activist groups. Thus, animal research will be a more familiar topic within our academic community. This conclusion also is supported by the high prevalence of identification in our survey of multiple sources of information on the subject within the last 5 years. We did not ask respondents to rank the importance of animal research relative to other issues, but can conclude that neither respondent population dismisses animal research as irrelevant, and that this issue merits targeted and proactive discussion on the university campus [2].

Our second research question sought to identify how much our respondents believed they knew about animal research. We asked for respondents’ confidence in their knowledge about arguments for and against animal research, and then about confidence in their knowledge about rules and regulations, about how well UW-Madison complied, and about adequacy of current regulations. Neither respondent group felt uniformly well-informed about any of these subjects. In 2014, over $200 million of research funding per year (about 20% of all extramural funding) at UW-Madison was associated with an Institutional Animal Care and Use Committee protocol, indicating that at least part of each of those funded projects was associated with animal use. Despite the magnitude of this funding, over 70% of students and 55% of the faculty indicated they knew only “some”, “a little”, or “nothing” about the “facts and arguments for and against the use of animals in research”.

Animal use in research in the United States, as elsewhere, must be managed by an Animal Care and Use Program [39] that is charged to ensure all relevant legal and regulatory requirements are met [40,41]. Lack of knowledge about those rules and regulations was also common among survey respondents: 47% of students and 19% of faculty answered “nothing” in response to the question “How much do you feel you know about the rules and regulations regarding the use and welfare of animals in research at UW-Madison?”. For questions asking about how well UW-Madison followed animal research laws and guidelines, 66–75% of students and 42–50% of faculty indicated “don’t know”. However, for those indicating knowledge, most respondents expressed confidence in the quality of compliance. Consistent with this widespread self-reported lack of knowledge about details, answers to our last question about knowledge indicated that 33% of faculty and 39% of students felt not at all to only a little confident that they had sufficient information to decide when or if animal research was acceptable. A survey of public attitudes in Great Britain toward animal research in 2016 reported a similar distribution of knowledge about use of animals in scientific research [28]. These findings highlight a tremendous gap in relevant knowledge about animal research within the university community, and point to an equally enormous opportunity to promote discussion of this controversial topic and improve the quality of personal and university decision-making.

Regarding our third general research questions about sources of information, students and faculty received information about animal research from a variety of sources, but for both groups UW-Madison courses and official spokespersons were most trusted to provide unbiased information. Furthermore, these two sources were most likely to be identified as providing too little information by both segments of the university community. The 2016 British survey of public attitudes [28] found animal care veterinarians and universities to be the most trusted sources to provide balanced information, followed by animal protection organizations. In our survey, animal “activist” groups were less trusted, but note the different choice of words. Fiske and Dupree [14] identify communicator credibility as composed of two elements: expertise (knowledge and accuracy) and trust. Trust, in particular, is accorded to scientists most often when they are viewed as teachers [14]. Teaching is embedded in university courses, and the trust expressed for UW-Madison spokespersons is consistent with their being viewed more as information-sharers than as advocates, even though their role does include advocacy. Of course, members of an organization may be predisposed to trust that organization compared to individuals on the outside. Yet regardless of cause, this trust will increase the willingness to engage in additional conversation. In fact, as mentioned above, our respondent populations expressed a strong desire for more such dialog.

Evaluations of demographic features of the responses allow us to refine our understanding of both student and faculty attitudes and their confidence in knowledge, and compare our respondent populations with those surveyed for other publications. A uniform finding in all studies that have looked at gender, including ours, is a greater prevalence of support among men for animal research [18–20,32,33,42–47]. We also observed that, among students, men found the issue of animal research less important than women, but, interestingly, this was not observed among faculty. With respect to knowledge of arguments, responses among students and faculty did not reflect a gender preference after correcting for other variables. Women students and faculty both felt slightly less confident than men in their ability to make informed decisions. The gender-dependence of support for animal research in our study will be addressed in more detail in a future publication. For all questions, faculty expressed greater interest, knowledge, and confidence in compliance and their own decision-making ability compared to students.

Academic discipline generally had a strong effect on answers to most questions, especially for faculty. Biology faculty reported that the issue was more important than did faculty in other disciplines. Strikingly, compared to biology faculty, physical sciences, social sciences, and the arts and humanities had odds ratios of 3, 8, and 19, respectively, regarding belief that the rules and regulations should be strengthened. Both biology students and faculty indicated greater knowledge of and ability to make decisions about the subject. Increased knowledge is not unexpected, since biology students and faculty are more likely to have learned about or experienced this inherently biological type of research. Although the influence of science education on attitudes toward animal research has been examined [32–34], we have not encountered a similarly detailed analysis of the influence of academic discipline.

Finally, pre-existing level of support for animal research had strong effects on rankings of importance, extent of knowledge, and belief in compliance. Importance also was influenced by animal experiences. Belief in compliance increased as support for animal research increased. Importance and knowledge about arguments generally were ranked higher by those either supporting or opposing animal research versus those with a neutral position. Thus, as expected for a controversial issue, people with strong feelings pro or con indicate greater importance of and confidence in knowledge about the subject. Perhaps most striking, though not unexpected, was the correlation of higher importance with respondents who had been a vegetarian or vegan or had worked on a research project using animals. This was especially marked for faculty, of which two-thirds of those who had worked with animals in research identified animal research as “extremely important”. Our findings support suggestions that people establish world-views about specific scientific issues characterized by consistency of beliefs [48].

In summary, students and faculty find the issue of animal research to be somewhat to very important, but display little confidence in their knowledge about how it is regulated. Among typical sources of information, UW-Madison courses and UW-Madison official spokespersons are most highly trusted to provide unbiased information, and both sources are viewed as providing too little information. Many in both respondent populations are “somewhat” to “a great deal” confident that they possess the necessary information to make decisions about the acceptability of animal research, though a large fraction (49% of students and 32% of faculty) are not.

### Implications of the history of animal research outreach

The history of extensive outreach about animal research at UW-Madison influences our conclusions about appropriate communication strategies and how best to foster good-faith dialog within the academic community. First, the trust given to UW-Madison sources of information may be in part a consequence of our historical pattern of open communication. Organizations with a more limited history of communication may need to build trust as they initiate their program of outreach. Second, even in the context of an environment with active communication about animal research, our university community has relatively little confidence in its understanding of how and how well animal research is regulated, and the general public almost certainly would be even less well-informed. Campus knowledge likely would have been more limited in the absence of past outreach. Making good decisions about when animal research is appropriate requires an understanding of the strengths and limitations of current regulations. This understanding is particularly lacking outside of the biological sciences for both students and faculty. Programs of outreach need to include discussions of current rules and regulations, and will be strengthened by honest and thorough discussions of the organization’s history of compliance coupled with an explicit recognition that being in compliance does not equate with being ethically justified. Third, because animal research is seen as a somewhat to extremely important issue to most students and even more so by faculty across all disciplines, efforts to explain the subject, pro-actively address concerns, and provide public- or community-wide opportunities for dialog from a diverse array of viewpoints should be viewed as a best practice in the university setting. Fourth, given that our survey found that students and faculty trust course-based information and UW-Madison spokespersons and that many students and faculty report hearing too little from these sources, our findings strongly support the conclusion that additional course-based discussion of the ethics of animal research and additional university community engagement by university spokespersons would be appreciated by our academic colleagues.

### Conclusion

Our observations indicate that UW-Madison student and faculty views about animal use in research display demographic variation commonly found in other surveys. Overall, animal research is identified as being an important issue, though not one that is well-understood. These findings suggest that, locally, UW-Madison should invest in additional campus outreach efforts to discuss the arguments for and against the use of animals in research, the details of regulation of this subject, and the effectiveness of compliance with the regulations. In particular, students and faculty outside of the biological sciences claim to have relatively little understanding of these subjects, and correspondingly feel less able to make informed decisions about when, how, or if at all animal research is acceptable. The suggestion to invest in additional outreach efforts should be appropriate at other institutions as well, and may be viewed as the reasonable default position when addressing other controversial topics. The university is trusted by many to be honest about this issue, but both students and faculty indicated that the university is providing too little information about these subjects. Outreach and support for public dialog about animal research should be considered best practices, and failure to meet these standards does a disservice to the university community.

## Supporting information

S1 Table Importance. (DOCX)

S2 Table Arguments. (DOCX)

S3 Table Rules and regulations. (DOCX)

S4 Table Enforcement. (DOCX)

S5 Table Review. (DOCX)

S6 Table Minimizing harm. (DOCX)

S7 Table Sufficiency. (DOCX)

S8 Table Informed decisions. (DOCX)
