# Supplementary material for: Assessing undergraduate student and faculty views on animal research: What do they know, whom do they trust, and how much do they care?
Source: PLoS One. 2019 Oct 24;14(10):e0223375. doi: 10.1371/journal.pone.0223375 (PMC6812826; doi:10.1371/journal.pone.0223375)
Supplement: S1 Table — (DOCX) [file pone.0223375.s001.docx]

| **S1 Table. Importance (1-5 scale)** | | | | | | | | | | | | | | | | | |
| --- | --- | --- | --- | --- | --- | --- | --- | --- | --- | --- | --- | --- | --- | --- | --- | --- | --- |
|  | Students | | | | | | | |  | Faculty | | | | | | | |
|  | Bivariate Analyses | | | |  | Multivariate Analyses | | |  | Bivariate Analyses | | | |  | Multivariate Analyses | | |
| Variables | Mean | SD | Value | p-value |  | Odds Ratio | | 95% CI |  | Mean | SD | Value | p-value |  | Odds Ratio | | 95% CI |
| Respondent characteristics |  |  |  |  |  |  |  |  |  |  |  |  |  |  |  |  |  |
| All | 3.2 | 1.0 |  |  |  |  |  |  |  | 3.6 | 1.2 |  |  |  |  |  |  |
|  |  |  |  |  |  |  |  |  |  |  |  |  |  |  |  |  |  |
| Gender |  |  |  |  |  |  |  |  |  |  |  |  |  |  |  |  |  |
| (Male) | 2.9 | 1.1 | -6.2 | .000 |  |  |  |  |  | 3.6 | 1.3 | .76 | .446 |  |  |  |  |
| Female | 3.4 | 1.0 |  |  |  | 2.3 | .001 | [1.4, 3.7] |  | 3.6 | 1.1 |  |  |  | .76 | .234 | [.49, 1.2] |
|  |  |  |  |  |  |  |  |  |  |  |  |  |  |  |  |  |  |
| Division |  |  |  |  |  |  |  |  |  |  |  |  |  |  |  |  |  |
| (Biological Sciences) | 3.4 | 1.0 | 17 | .001 |  |  |  |  |  | 4.3 | 1.0 | 218 | .000 |  |  |  |  |
| Physical Sciences | 3.0 | 1.0 |  |  |  | .82 | .470 | [.48, 1.4] |  | 3.1 | 1.2 |  |  |  | .11 | .000 | [.08, .17] |
| Social Sciences | 3.1 | 1.0 |  |  |  | .85 | .610 | [.46, 1.6] |  | 3.1 | 1.1 |  |  |  | .10 | .000 | [.06, .15] |
| Humanities | 3.3 | 1.0 |  |  |  | 1.1 | .815 | [.45, 2.8] |  | 3.2 | 1.0 |  |  |  | .14 | .000 | [.09, .22] |
|  |  |  |  |  |  |  |  |  |  |  |  |  |  |  |  |  |  |
| Year in School |  |  |  |  |  |  |  |  |  |  |  |  |  |  |  |  |  |
| (Freshman) | 3.2 | 1.0 | 1.3 | .719 |  |  |  |  |  |  |  |  |  |  |  |  |  |
| Sophomore | 3.2 | 1.0 |  |  |  | 1.1 | .619 | [.75, 1.6] |  |  |  |  |  |  |  |  |  |
| Junior | 3.3 | 1.0 |  |  |  | 1.4 | .070 | [.97, 2.0] |  |  |  |  |  |  |  |  |  |
| Senior | 3.2 | 1.1 |  |  |  | 1.2 | .265 | [.85, 1.8] |  |  |  |  |  |  |  |  |  |
|  |  |  |  |  |  |  |  |  |  |  |  |  |  |  |  |  |  |
| Academic Rank |  |  |  |  |  |  |  |  |  |  |  |  |  |  |  |  |  |
| (Assistant Professor) |  |  |  |  |  |  |  |  |  | 3.4 | 1.2 | 3.0 | .220 |  |  |  |  |
| Associate Professor |  |  |  |  |  |  |  |  |  | 3.6 | 1.1 |  |  |  | 1.3 | .199 | [.88, 1.9] |
| Full Professor |  |  |  |  |  |  |  |  |  | 3.6 | 1.2 |  |  |  | 1.3 | .116 | [.94, 1.7] |
|  |  |  |  |  |  |  |  |  |  |  |  |  |  |  |  |  |  |
| QIVB Category |  |  |  |  |  |  |  |  |  |  |  |  |  |  |  |  |  |
| (Neither agree nor disagree) | 2.9 | 0.8 | 90 | .000 |  |  |  |  |  | 3.1 | 1.0 | 49 | .000 |  |  |  |  |
| Agree or Strongly Agree | 3.0 | 1.1 |  |  |  | 1.2 | .368 | [.83, 1.7] |  | 3.7 | 1.2 |  |  |  | 2.1 | .000 | [1.5, 2.9] |
| Disagree or Strongly Disagree | 3.7 | 0.9 |  |  |  | 4.3 | .000 | [3.0, 6.2] |  | 3.6 | 1.0 |  |  |  | 2.4 | .000 | [1.7, 3.5] |
|  |  |  |  |  |  |  |  |  |  |  |  |  |  |  |  |  |  |
| Interaction Terms (If Significant) |  |  |  |  |  |  |  |  |  |  |  |  |  |  |  |  |  |
| Female X Humanities |  |  |  |  |  | .57 | .320 | [.19, 1.7] |  |  |  |  |  |  | 1.3 | .415 | [.66, 2.7] |
| Female X Physical Sciences |  |  |  |  |  | .58 | .188 | [.26, 1.3] |  |  |  |  |  |  | 1.5 | .389 | [.62, 3.5] |
| Female X Social Sciences |  |  |  |  |  | .60 | .178 | [.29, 1.3] |  |  |  |  |  |  | 2.3 | .014 | [1.2, 4.3] |
|  |  |  |  |  |  |  |  |  |  |  |  |  |  |  |  |  |  |
| Model fit statistics |  |  |  |  |  |  |  |  |  |  |  |  |  |  |  |  |  |
| N |  |  |  |  |  | 738 |  |  |  |  |  |  |  |  | 938 |  |  |
| Pseudo R2 |  |  |  |  |  | .0603 |  |  |  |  |  |  |  |  | .1007 |  |  |
| Log likelihood |  |  |  |  |  | -988 |  |  |  |  |  |  |  |  | -1244 |  |  |

Bivariate analyses for binary variables are Wilcoxon/Mann-Whitney tests while non-binary variables are Kruskal-Wallis tests.
