# Supplementary material for: Assessing undergraduate student and faculty views on animal research: What do they know, whom do they trust, and how much do they care?
Source: PLoS One. 2019 Oct 24;14(10):e0223375. doi: 10.1371/journal.pone.0223375 (PMC6812826; doi:10.1371/journal.pone.0223375)
Supplement: S2 Table — (DOCX) [file pone.0223375.s002.docx]

| **S2 Table. Arguments (1-5 scale)** | | | | | | | | | | | | | | | | | |
| --- | --- | --- | --- | --- | --- | --- | --- | --- | --- | --- | --- | --- | --- | --- | --- | --- | --- |
|  | Students | | | | | | | |  | Faculty | | | | | | | |
|  | Bivariate Analyses | | | |  | Multivariate Analyses | | |  | Bivariate Analyses | | | |  | Multivariate Analyses | | |
| Variables | Mean | SD | Value | p-value |  | Odds Ratio | | 95% CI |  | Mean | SD | Value | p-value |  | Odds Ratio | | 95% CI |
| Respondent characteristics |  |  |  |  |  |  |  |  |  |  |  |  |  |  |  |  |  |
| All | 3.0 | .96 |  |  |  |  |  |  |  | 3.4 | 1.1 |  |  |  |  |  |  |
|  |  |  |  |  |  |  |  |  |  |  |  |  |  |  |  |  |  |
| Gender |  |  |  |  |  |  |  |  |  |  |  |  |  |  |  |  |  |
| (Male) | 2.9 | .96 | -1.0 | .299 |  |  |  |  |  | 3.5 | 1.1 | 3.1 | .002 |  |  |  |  |
| Female | 3.0 | .94 |  |  |  | .92 | .732 | [.58, 1.5] |  | 3.2 | .95 |  |  |  | .66 | .057 | [.43, 1.0] |
|  |  |  |  |  |  |  |  |  |  |  |  |  |  |  |  |  |  |
| Division |  |  |  |  |  |  |  |  |  |  |  |  |  |  |  |  |  |
| (Biological Sciences) | 3.2 | .95 | 41 | .000 |  |  |  |  |  | 4.0 | .87 | 247 | .000 |  |  |  |  |
| Physical Sciences | 2.8 | .91 |  |  |  | .41 | .001 | [.24, .69] |  | 2.9 | 1.0 |  |  |  | .09 | .000 | [.06, .14] |
| Social Sciences | 2.8 | .87 |  |  |  | .27 | .000 | [.15, .50] |  | 3.0 | .91 |  |  |  | .14 | .000 | [.09, .22] |
| Humanities | 2.9 | .89 |  |  |  | .84 | .702 | [.34, 2.1] |  | 2.9 | .85 |  |  |  | .12 | .000 | [.08, .19] |
|  |  |  |  |  |  |  |  |  |  |  |  |  |  |  |  |  |  |
| Year in School |  |  |  |  |  |  |  |  |  |  |  |  |  |  |  |  |  |
| (Freshman) | 2.85 | .95 | 12 | .008 |  |  |  |  |  |  |  |  |  |  |  |  |  |
| Sophomore | 2.9 | .91 |  |  |  | 1.0 | .965 | [.69, 1.5] |  |  |  |  |  |  |  |  |  |
| Junior | 3.1 | .94 |  |  |  | 1.6 | .014 | [1.1, 2.3] |  |  |  |  |  |  |  |  |  |
| Senior | 3.1 | 1.0 |  |  |  | 1.6 | .025 | [1.1, 2.3] |  |  |  |  |  |  |  |  |  |
|  |  |  |  |  |  |  |  |  |  |  |  |  |  |  |  |  |  |
| Academic Rank |  |  |  |  |  |  |  |  |  |  |  |  |  |  |  |  |  |
| (Assistant Professor) |  |  |  |  |  |  |  |  |  | 3.2 | 1.1 | 12 | .002 |  |  |  |  |
| Associate Professor |  |  |  |  |  |  |  |  |  | 3.4 | .98 |  |  |  | 1.4 | .100 | [.94, 2.0] |
| Full Professor |  |  |  |  |  |  |  |  |  | 3.5 | 1.1 |  |  |  | 1.6 | .006 | [1.1, 2.1] |
|  |  |  |  |  |  |  |  |  |  |  |  |  |  |  |  |  |  |
| QIVB Category |  |  |  |  |  |  |  |  |  |  |  |  |  |  |  |  |  |
| (Neither agree nor disagree) | 2.6 | .85 | 41 | .000 |  |  |  |  |  | 2.9 | .90 | 65 | .000 |  |  |  |  |
| Agree or Strongly Agree | 3.2 | .99 |  |  |  | 2.9 | .000 | [2.0, 4.2] |  | 3.6 | 1.1 |  |  |  | 2.4 | .000 | [1.7, 3.3] |
| Disagree or Strongly Disagree | 3.0 | .91 |  |  |  | 2.5 | .000 | [1.7, 3.6] |  | 3.2 | 1.0 |  |  |  | 1.9 | .001 | [1.3, 2.8] |
|  |  |  |  |  |  |  |  |  |  |  |  |  |  |  |  |  |  |
| Interaction Terms (If Significant) |  |  |  |  |  |  |  |  |  |  |  |  |  |  |  |  |  |
| Female X Humanities |  |  |  |  |  | .48 | .196 | [.16, 1.5] |  |  |  |  |  |  | 1.0 | .924 | [.51, 2.1] |
| Female X Physical Sciences |  |  |  |  |  | .82 | .624 | [.37, 1.8] |  |  |  |  |  |  | 1.8 | .176 | [.76, 4.4] |
| Female X Social Sciences |  |  |  |  |  | 1.5 | .254 | [.74, 3.2] |  |  |  |  |  |  | 1.4 | .323 | [.73, 2.6] |
|  |  |  |  |  |  |  |  |  |  |  |  |  |  |  |  |  |  |
| Model fit statistics |  |  |  |  |  |  |  |  |  |  |  |  |  |  |  |  |  |
| N |  |  |  |  |  | 738 |  |  |  |  |  |  |  |  | 939 |  |  |
| Pseudo R2 |  |  |  |  |  | .0482 |  |  |  |  |  |  |  |  | .1179 |  |  |
| Log likelihood |  |  |  |  |  | -948 |  |  |  |  |  |  |  |  | -1178 |  |  |

Bivariate analyses for binary variables are Wilcoxon/Mann-Whitney tests while non-binary variables are Kruskal-Wallis tests.
