# Supplementary material for: Assessing undergraduate student and faculty views on animal research: What do they know, whom do they trust, and how much do they care?
Source: PLoS One. 2019 Oct 24;14(10):e0223375. doi: 10.1371/journal.pone.0223375 (PMC6812826; doi:10.1371/journal.pone.0223375)
Supplement: S3 Table — (DOCX) [file pone.0223375.s003.docx]

| **S3 Table. Rules and Regulations, % don’t know** | | | | | | | | | | | | | | | |
| --- | --- | --- | --- | --- | --- | --- | --- | --- | --- | --- | --- | --- | --- | --- | --- |
|  | Students | | | | | | |  | Faculty | | | | | | |
|  | Bivariate Analyses | | |  | Multivariate Analyses | | |  | Bivariate Analyses | | |  | Multivariate Analyses | | |
| Variables | Proportion | Value | p-value |  | Odds Ratio | | 95% CI |  | Proportion | Value | p-value |  | Odds Ratio | | 95% CI |
| Respondent characteristics |  |  |  |  |  |  |  |  |  |  |  |  |  |  |  |
| All | 47 |  |  |  |  |  |  |  | 19 |  |  |  |  |  |  |
|  |  |  |  |  |  |  |  |  |  |  |  |  |  |  |  |
| Gender |  |  |  |  |  |  |  |  |  |  |  |  |  |  |  |
| (Male) | 49 | .71 | .476 |  |  |  |  |  | 18 | -2.0 | .046 |  |  |  |  |
| Female | 46 |  |  |  | 1.3 | .364 | [.75, 2.2] |  | 23 |  |  |  | .51 | .544 | [.06, 4.5] |
|  |  |  |  |  |  |  |  |  |  |  |  |  |  |  |  |
| Division |  |  |  |  |  |  |  |  |  |  |  |  |  |  |  |
| (Biological Sciences) | 37 | 22 | .000 |  |  |  |  |  | 2 | 128 | .000 |  |  |  |  |
| Physical Sciences | 56 |  |  |  | 2.4 | .005 | [1.3, 4.3] |  | 32 |  |  |  | 29 | .000 | [11, 77] |
| Social Sciences | 55 |  |  |  | 3.2 | .001 | [1.6, 6.6] |  | 24 |  |  |  | 14 | .000 | [5.2, 38] |
| Humanities | 46 |  |  |  | 2.9 | .040 | [1.1, 8.0] |  | 36 |  |  |  | 20 | .000 | [7.3, 54] |
|  |  |  |  |  |  |  |  |  |  |  |  |  |  |  |  |
| Year in School |  |  |  |  |  |  |  |  |  |  |  |  |  |  |  |
| (Freshman) | 53 | 13 | .006 |  |  |  |  |  |  |  |  |  |  |  |  |
| Sophomore | 53 |  |  |  | 1.0 | .916 | [.67, 1.6] |  |  |  |  |  |  |  |  |
| Junior | 43 |  |  |  | .70 | .092 | [.47, 1.1] |  |  |  |  |  |  |  |  |
| Senior | 38 |  |  |  | .56 | .007 | [.37, .86] |  |  |  |  |  |  |  |  |
|  |  |  |  |  |  |  |  |  |  |  |  |  |  |  |  |
| Academic Rank |  |  |  |  |  |  |  |  |  |  |  |  |  |  |  |
| (Assistant Professor) |  |  |  |  |  |  |  |  | 33 | 29 | .000 |  |  |  |  |
| Associate Professor |  |  |  |  |  |  |  |  | 16 |  |  |  | .36 | .000 | [.21, .63] |
| Full Professor |  |  |  |  |  |  |  |  | 16 |  |  |  | .35 | .000 | [.23, .53] |
|  |  |  |  |  |  |  |  |  |  |  |  |  |  |  |  |
| QIVB Category |  |  |  |  |  |  |  |  |  |  |  |  |  |  |  |
| (Neither agree nor disagree) | 51 | 8.0 | .019 |  |  |  |  |  | 25 | 19 | .000 |  |  |  |  |
| Agree or Strongly Agree | 41 |  |  |  | .74 | .151 | [.50, 1.1] |  | 15 |  |  |  | .80 | .326 | [.50, 1.3] |
| Disagree or Strongly Disagree | 51 |  |  |  | 1.1 | .763 | [.71, 1.6] |  | 28 |  |  |  | 1.1 | .716 | [.66, 1.8] |
|  |  |  |  |  |  |  |  |  |  |  |  |  |  |  |  |
| Interaction Terms (If Significant) |  |  |  |  |  |  |  |  |  |  |  |  |  |  |  |
| Female X Humanities |  |  |  |  | .35 | .095 | [.10, 1.2] |  |  |  |  |  | 3.8 | .246 | [.40, 37] |
| Female X Physical Sciences |  |  |  |  | 1.4 | .485 | [.55, 3.5] |  |  |  |  |  | 1.1 | .957 | [.10, 11] |
| Female X Social Sciences |  |  |  |  | .52 | .127 | [.22, 1.2] |  |  |  |  |  | 2.2 | .491 | [.23, 20] |
|  |  |  |  |  |  |  |  |  |  |  |  |  |  |  |  |
| Model fit statistics |  |  |  |  |  |  |  |  |  |  |  |  |  |  |  |
| N |  |  |  |  | 738 |  |  |  |  |  |  |  | 940 |  |  |
| Pseudo R2 |  |  |  |  | .0475 |  |  |  |  |  |  |  | .2104 |  |  |
| Log likelihood |  |  |  |  | -485 |  |  |  |  |  |  |  | -365 |  |  |

Bivariate analyses for binary variables are pr-tests while non-binary variables are Chi2 tests.

| **Rules and Regulations, with an opinion (2-5 scale)** | | | | | | | | | | | | | | | | | |
| --- | --- | --- | --- | --- | --- | --- | --- | --- | --- | --- | --- | --- | --- | --- | --- | --- | --- |
|  | Students | | | | | | | |  | Faculty | | | | | | | |
|  | Bivariate Analyses | | | |  | Multivariate Analyses | | |  | Bivariate Analyses | | | |  | Multivariate Analyses | | |
| Variables | Mean | SD | Value | p-value |  | Odds Ratio | | 95% CI |  | Mean | SD | Value | p-value |  | Odds Ratio | | 95% CI |
| Respondent characteristics |  |  |  |  |  |  |  |  |  |  |  |  |  |  |  |  |  |
| All | 2.69 | .89 |  |  |  |  |  |  |  | 3.2 | 1.1 |  |  |  |  |  |  |
|  |  |  |  |  |  |  |  |  |  |  |  |  |  |  |  |  |  |
| Gender |  |  |  |  |  |  |  |  |  |  |  |  |  |  |  |  |  |
| (Male) | 2.69 | .91 | .03 | .980 |  |  |  |  |  | 3.3 | 1.1 | 2.6 | .010 |  |  |  |  |
| Female | 2.67 | .87 |  |  |  | 1.1 | .812 | [.60, 1.9] |  | 3.0 | .99 |  |  |  | .67 | .073 | [.43, 1.0] |
|  |  |  |  |  |  |  |  |  |  |  |  |  |  |  |  |  |  |
| Division |  |  |  |  |  |  |  |  |  |  |  |  |  |  |  |  |  |
| (Biological Sciences) | 2.89 | .97 | 26 | .000 |  |  |  |  |  | 3.75 | 1.0 | 193 | .000 |  |  |  |  |
| Physical Sciences | 2.44 | .75 |  |  |  | .42 | .019 | [.20, .87] |  | 2.78 | .91 |  |  |  | .12 | .000 | [.08, .20] |
| Social Sciences | 2.38 | .61 |  |  |  | .39 | .059 | [.15, 1.0] |  | 2.69 | .80 |  |  |  | .14 | .000 | [.09, .22] |
| Humanities | 2.60 | .97 |  |  |  | .74 | .709 | [.15, 3.7] |  | 2.57 | .69 |  |  |  | .11 | .000 | [.40, 1.8] |
|  |  |  |  |  |  |  |  |  |  |  |  |  |  |  |  |  |  |
| Year in School |  |  |  |  |  |  |  |  |  |  |  |  |  |  |  |  |  |
| (Freshman) | 2.56 | .79 | 5.7 | .127 |  |  |  |  |  |  |  |  |  |  |  |  |  |
| Sophomore | 2.63 | .78 |  |  |  | 1.1 | .789 | [.59, 2.0] |  |  |  |  |  |  |  |  |  |
| Junior | 2.63 | .83 |  |  |  | 1.0 | .987 | [.57, 1.8] |  |  |  |  |  |  |  |  |  |
| Senior | 2.90 | 1.1 |  |  |  | 1.7 | .061 | [.98, 3.0] |  |  |  |  |  |  |  |  |  |
|  |  |  |  |  |  |  |  |  |  |  |  |  |  |  |  |  |  |
| Academic Rank |  |  |  |  |  |  |  |  |  |  |  |  |  |  |  |  |  |
| (Assistant Professor) |  |  |  |  |  |  |  |  |  | 3.1 | 1.1 | 3.9 | .145 |  |  |  |  |
| Associate Professor |  |  |  |  |  |  |  |  |  | 3.1 | 1.0 |  |  |  | 1.1 | .804 | [.68, 1.7] |
| Full Professor |  |  |  |  |  |  |  |  |  | 3.2 | 1.1 |  |  |  | 1.4 | .087 | [.95, 2.0] |
|  |  |  |  |  |  |  |  |  |  |  |  |  |  |  |  |  |  |
| QIVB Category |  |  |  |  |  |  |  |  |  |  |  |  |  |  |  |  |  |
| (Neither agree nor disagree) | 2.40 | .61 | 27 | .000 |  |  |  |  |  | 2.7 | .85 | 67 | .000 |  |  |  |  |
| Agree or Strongly Agree | 2.94 | 1.0 |  |  |  | 2.6 | .001 | [1.5, 4.6] |  | 3.4 | 1.1 |  |  |  | 3.0 | .000 | [2.0, 4.4] |
| Disagree or Strongly Disagree | 2.48 | .73 |  |  |  | 1.1 | .658 | [.63, 2.1] |  | 2.9 | .93 |  |  |  | 1.8 | .012 | [1.1, 2.9] |
|  |  |  |  |  |  |  |  |  |  |  |  |  |  |  |  |  |  |
| Interaction Terms (If Significant) |  |  |  |  |  |  |  |  |  |  |  |  |  |  |  |  |  |
| Female X Humanities |  |  |  |  |  | .66 | .665 | [.10, 4.3] |  |  |  |  |  |  | 1.5 | .375 | [.62, 3.6] |
| Female X Physical Sciences |  |  |  |  |  | .17 | .120 | [.02, 1.6] |  |  |  |  |  |  | 2.1 | .136 | [.79, 5.8] |
| Female X Social Sciences |  |  |  |  |  | .98 | .970 | [.31, 3.1] |  |  |  |  |  |  | 1.4 | .404 | [.66, 2.8] |
|  |  |  |  |  |  |  |  |  |  |  |  |  |  |  |  |  |  |
| Model fit statistics |  |  |  |  |  |  |  |  |  |  |  |  |  |  |  |  |  |
| N |  |  |  |  |  | 397 |  |  |  |  |  |  |  |  | 757 |  |  |
| Pseudo R2 |  |  |  |  |  | .0667 |  |  |  |  |  |  |  |  | .1256 |  |  |
| Log likelihood |  |  |  |  |  | -409 |  |  |  |  |  |  |  |  | -888 |  |  |

Bivariate analyses for binary variables are Wilcoxon/Mann-Whitney tests while non-binary variables are Kruskal-Wallis tests.
