# Supplementary material for: Assessing undergraduate student and faculty views on animal research: What do they know, whom do they trust, and how much do they care?
Source: PLoS One. 2019 Oct 24;14(10):e0223375. doi: 10.1371/journal.pone.0223375 (PMC6812826; doi:10.1371/journal.pone.0223375)
Supplement: S4 Table — (DOCX) [file pone.0223375.s004.docx]

| **S4 Table. Enforcement, % don’t know** | | | | | | | | | | | | | | | |
| --- | --- | --- | --- | --- | --- | --- | --- | --- | --- | --- | --- | --- | --- | --- | --- |
|  | Students | | | | | | |  | Faculty | | | | | | |
|  | Bivariate Analyses | | |  | Multivariate Analyses | | |  | Bivariate Analyses | | |  | Multivariate Analyses | | |
| Variables | Proportion | Value | p-value |  | Odds Ratio | | 95% CI |  | Proportion | Value | p-value |  | Odds Ratio | | 95% CI |
| Respondent characteristics |  |  |  |  |  |  |  |  |  |  |  |  |  |  |  |
| All | 75 |  |  |  |  |  |  |  | 47 |  |  |  |  |  |  |
|  |  |  |  |  |  |  |  |  |  |  |  |  |  |  |  |
| Gender |  |  |  |  |  |  |  |  |  |  |  |  |  |  |  |
| (Male) | 76 | .31 | .756 |  |  |  |  |  | 42 | -3.6 | .000 |  |  |  |  |
| Female | 75 |  |  |  | 1.0 | .942 | [.59, 1.8] |  | 55 |  |  |  | 2.1 | .007 | [1.2, 3.7] |
|  |  |  |  |  |  |  |  |  |  |  |  |  |  |  |  |
| Division |  |  |  |  |  |  |  |  |  |  |  |  |  |  |  |
| (Biological Sciences) | 68 | 23 | .000 |  |  |  |  |  | 22 | 145 | .000 |  |  |  |  |
| Physical Sciences | 86 |  |  |  | 3.0 | .002 | [1.5, 5.9] |  | 57 |  |  |  | 5.8 | .000 | [3.7, 9.2] |
| Social Sciences | 81 |  |  |  | 1.6 | .221 | [.75, 3.5] |  | 65 |  |  |  | 7.6 | .000 | [4.6, 13] |
| Humanities | 77 |  |  |  | 1.2 | .778 | [.40, 3.5] |  | 63 |  |  |  | 6.2 | .000 | [3.6, 11] |
|  |  |  |  |  |  |  |  |  |  |  |  |  |  |  |  |
| Year in School |  |  |  |  |  |  |  |  |  |  |  |  |  |  |  |
| (Freshman) | 79 | 6.8 | .080 |  |  |  |  |  |  |  |  |  |  |  |  |
| Sophomore | 77 |  |  |  | .90 | .679 | [.53, 1.5] |  |  |  |  |  |  |  |  |
| Junior | 75 |  |  |  | .76 | .284 | [.47, 1.3] |  |  |  |  |  |  |  |  |
| Senior | 68 |  |  |  | .52 | .008 | [.32, .85] |  |  |  |  |  |  |  |  |
|  |  |  |  |  |  |  |  |  |  |  |  |  |  |  |  |
| Academic Rank |  |  |  |  |  |  |  |  |  |  |  |  |  |  |  |
| (Assistant Professor) |  |  |  |  |  |  |  |  | 60 | 21 | .000 |  |  |  |  |
| Associate Professor |  |  |  |  |  |  |  |  | 49 |  |  |  | .68 | .097 | [.43, 1.1] |
| Full Professor |  |  |  |  |  |  |  |  | 41 |  |  |  | .48 | .000 | [.33, .70] |
|  |  |  |  |  |  |  |  |  |  |  |  |  |  |  |  |
| Q3b Category |  |  |  |  |  |  |  |  |  |  |  |  |  |  |  |
| (Neither agree nor disagree) | 81 | 12 | .002 |  |  |  |  |  | 62 | 65 | .000 |  |  |  |  |
| Agree or Strongly Agree | 69 |  |  |  | .48 | .004 | [.29, .79] |  | 36 |  |  |  | .45 | .000 | [.31, .66] |
| Disagree or Strongly Disagree | 79 |  |  |  | .84 | .508 | [.49, 1.4] |  | 64 |  |  |  | .96 | .847 | [.60, 1.5] |
|  |  |  |  |  |  |  |  |  |  |  |  |  |  |  |  |
| Interaction Terms (If Significant) |  |  |  |  |  |  |  |  |  |  |  |  |  |  |  |
| Female X Humanities |  |  |  |  | 1.4 | .612 | [.36, 5.8] |  |  |  |  |  | .42 | .047 | [.18, .99] |
| Female X Physical Sciences |  |  |  |  | 1.4 | .644 | [.38, 4.8] |  |  |  |  |  | .65 | .411 | [.23, 1.8] |
| Female X Social Sciences |  |  |  |  | 1.3 | .569 | [.51, 3.5] |  |  |  |  |  | .44 | .041 | [.20, .97] |
|  |  |  |  |  |  |  |  |  |  |  |  |  |  |  |  |
| Model fit statistics |  |  |  |  |  |  |  |  |  |  |  |  |  |  |  |
| N |  |  |  |  | 738 |  |  |  |  |  |  |  | 940 |  |  |
| Pseudo R2 |  |  |  |  | .0602 |  |  |  |  |  |  |  | .1616 |  |  |
| Log likelihood |  |  |  |  | -384 |  |  |  |  |  |  |  | -545 |  |  |

Bivariate analyses for binary variables are pr-tests while non-binary variables are Chi2 tests.

| **Enforcement, with an opinion (1-5 scale)** | | | | | | | | | | | | | | | | | |
| --- | --- | --- | --- | --- | --- | --- | --- | --- | --- | --- | --- | --- | --- | --- | --- | --- | --- |
|  | Students | | | | | | | |  | Faculty | | | | | | | |
|  | Bivariate Analyses | | | |  | Multivariate Analyses | | |  | Bivariate Analyses | | | |  | Multivariate Analyses | | |
| Variables | Mean | SD | Value | p-value |  | Odds Ratio | | 95% CI |  | Mean | SD | Value | p-value |  | Odds Ratio | | 95% CI |
| Respondent characteristics |  |  |  |  |  |  |  |  |  |  |  |  |  |  |  |  |  |
| All | 37 | 1.0 |  |  |  |  |  |  |  | 4.3 | .72 |  |  |  |  |  |  |
|  |  |  |  |  |  |  |  |  |  |  |  |  |  |  |  |  |  |
| Gender |  |  |  |  |  |  |  |  |  |  |  |  |  |  |  |  |  |
| (Male) | 3.9 | .93 | 1.0 | .316 |  |  |  |  |  | 4.3 | .67 | 1.6 | .114 |  |  |  |  |
| Female | 3.7 | 1.0 |  |  |  | .57 | .189 | [.25, 1.3] |  | 4.2 | .81 |  |  |  | .59 | .062 | [.33, 1.0] |
|  |  |  |  |  |  |  |  |  |  |  |  |  |  |  |  |  |  |
| Division |  |  |  |  |  |  |  |  |  |  |  |  |  |  |  |  |  |
| (Biological Sciences) | 4.0 | .83 | 18 | .000 |  |  |  |  |  | 4.5 | .57 | 51 | .000 |  |  |  |  |
| Physical Sciences | 3.7 | .70 |  |  |  | .26 | .026 | [.08, .85] |  | 4.2 | .58 |  |  |  | .36 | .000 | [.21, .62] |
| Social Sciences | 3.3 | 1.2 |  |  |  | .34 | .107 | [.09, 1.3] |  | 4.1 | .86 |  |  |  | .36 | .002 | [.19, .69] |
| Humanities | 2.8 | 1.3 |  |  |  | .17 | .073 | [.02, 1.2] |  | 3.7 | .90 |  |  |  | .09 | .000 | [.04, .19] |
|  |  |  |  |  |  |  |  |  |  |  |  |  |  |  |  |  |  |
| Year in School |  |  |  |  |  |  |  |  |  |  |  |  |  |  |  |  |  |
| (Freshman) | 3.4 | 1.1 | 8.7 | .034 |  |  |  |  |  |  |  |  |  |  |  |  |  |
| Sophomore | 3.6 | .93 |  |  |  | 1.1 | .782 | [.45, 2.9] |  |  |  |  |  |  |  |  |  |
| Junior | 3.9 | .94 |  |  |  | 1.6 | .318 | [.64, 4.0] |  |  |  |  |  |  |  |  |  |
| Senior | 3.9 | 1.0 |  |  |  | 1.8 | .175 | [.77, 4.4] |  |  |  |  |  |  |  |  |  |
|  |  |  |  |  |  |  |  |  |  |  |  |  |  |  |  |  |  |
| Academic Rank |  |  |  |  |  |  |  |  |  |  |  |  |  |  |  |  |  |
| (Assistant Professor) |  |  |  |  |  |  |  |  |  | 4.4 | .65 | 3.8 | .148 |  |  |  |  |
| Associate Professor |  |  |  |  |  |  |  |  |  | 4.1 | .83 |  |  |  | .47 | .018 | [.26, .88] |
| Full Professor |  |  |  |  |  |  |  |  |  | 4.3 | .69 |  |  |  | .74 | .255 | [.45, 1.2] |
|  |  |  |  |  |  |  |  |  |  |  |  |  |  |  |  |  |  |
| QIVB Category |  |  |  |  |  |  |  |  |  |  |  |  |  |  |  |  |  |
| (Neither agree nor disagree) | 3.6 | 1.0 | 39 | .000 |  |  |  |  |  | 4.0 | .68 | 37 | .000 |  |  |  |  |
| Agree or Strongly Agree | 4.1 | .84 |  |  |  | 1.5 | .411 | [.58, 3.7] |  | 4.4 | .63 |  |  |  | 2.6 | .000 | [1.6, 4.5] |
| Disagree or Strongly Disagree | 3.1 | .99 |  |  |  | .25 | .006 | [.10, .67] |  | 3.9 | .92 |  |  |  | .74 | .377 | [.37, 1.5] |
|  |  |  |  |  |  |  |  |  |  |  |  |  |  |  |  |  |  |
| Interaction Terms (If Significant) |  |  |  |  |  |  |  |  |  |  |  |  |  |  |  |  |  |
| Female X Humanities |  |  |  |  |  | .83 | .876 | [.08, 9.1] |  |  |  |  |  |  | 4.6 | .013 | [1.4, 15] |
| Female X Physical Sciences |  |  |  |  |  | 3.6 | .253 | [.40, 32] |  |  |  |  |  |  | 3.3 | .101 | [.79, 14] |
| Female X Social Sciences |  |  |  |  |  | 1.2 | .856 | [.23, 6.0] |  |  |  |  |  |  | 2.0 | .208 | [.68, 5.7] |
|  |  |  |  |  |  |  |  |  |  |  |  |  |  |  |  |  |  |
| Model fit statistics |  |  |  |  |  |  |  |  |  |  |  |  |  |  |  |  |  |
| N |  |  |  |  |  | 179 |  |  |  |  |  |  |  |  | 501 |  |  |
| Pseudo R2 |  |  |  |  |  | .1288 |  |  |  |  |  |  |  |  | .1026 |  |  |
| Log likelihood |  |  |  |  |  | -204 |  |  |  |  |  |  |  |  | -452 |  |  |

Bivariate analyses for binary variables are Wilcoxon/Mann-Whitney tests while non-binary variables are Kruskal-Wallis tests.
