# Supplementary material for: Assessing undergraduate student and faculty views on animal research: What do they know, whom do they trust, and how much do they care?
Source: PLoS One. 2019 Oct 24;14(10):e0223375. doi: 10.1371/journal.pone.0223375 (PMC6812826; doi:10.1371/journal.pone.0223375)
Supplement: S6 Table — (DOCX) [file pone.0223375.s006.docx]

| **S6 Table. Minimizing Harm, % don’t know** | | | | | | | | | | | | | | | |
| --- | --- | --- | --- | --- | --- | --- | --- | --- | --- | --- | --- | --- | --- | --- | --- |
|  | Students | | | | | | |  | Faculty | | | | | | |
|  | Bivariate Analyses | | |  | Multivariate Analyses | | |  | Bivariate Analyses | | |  | Multivariate Analyses | | |
| Variables | Proportion | Value | p-value |  | Odds Ratio | | 95% CI |  | Proportion | Value | p-value |  | Odds Ratio | | 95% CI |
| Respondent characteristics |  |  |  |  |  |  |  |  |  |  |  |  |  |  |  |
| All | 71 |  |  |  |  |  |  |  | 50 |  |  |  |  |  |  |
|  |  |  |  |  |  |  |  |  |  |  |  |  |  |  |  |
| Gender |  |  |  |  |  |  |  |  |  |  |  |  |  |  |  |
| (Male) | 74 | .70 | .481 |  |  |  |  |  | 48 | -1.7 | .099 |  |  |  |  |
| Female | 71 |  |  |  | .72 | .231 | [.42, 1.2] |  | 54 |  |  |  | 1.7 | .071 | [.96, 2.9] |
|  |  |  |  |  |  |  |  |  |  |  |  |  |  |  |  |
| Division |  |  |  |  |  |  |  |  |  |  |  |  |  |  |  |
| (Biological Sciences) | 63 | 23 | .000 |  |  |  |  |  | 22 | 189 | .000 |  |  |  |  |
| Physical Sciences | 80 |  |  |  | 1.7 | .090 | [.92, 3.3] |  | 69 |  |  |  | 10 | .000 | [6.5, 17] |
| Social Sciences | 78 |  |  |  | 1.3 | .531 | [.60, 2.7] |  | 67 |  |  |  | 8.6 | .000 | [5.2, 14] |
| Humanities | 73 |  |  |  | 1.2 | .709 | [.41, 3.8] |  | 69 |  |  |  | 8.3 | .000 | [4.8, 14] |
|  |  |  |  |  |  |  |  |  |  |  |  |  |  |  |  |
| Year in School |  |  |  |  |  |  |  |  |  |  |  |  |  |  |  |
| (Freshman) | 79 | 13 | .005 |  |  |  |  |  |  |  |  |  |  |  |  |
| Sophomore | 74 |  |  |  | .81 | .422 | [.49, 1.3] |  |  |  |  |  |  |  |  |
| Junior | 66 |  |  |  | .51 | .005 | [.32, .82] |  |  |  |  |  |  |  |  |
| Senior | 66 |  |  |  | .50 | .004 | [.31, .81] |  |  |  |  |  |  |  |  |
|  |  |  |  |  |  |  |  |  |  |  |  |  |  |  |  |
| Academic Rank |  |  |  |  |  |  |  |  |  |  |  |  |  |  |  |
| (Assistant Professor) |  |  |  |  |  |  |  |  | 62 | 16 | .000 |  |  |  |  |
| Associate Professor |  |  |  |  |  |  |  |  | 53 |  |  |  | .78 | .296 | [.48, 1.2] |
| Full Professor |  |  |  |  |  |  |  |  | 46 |  |  |  | .49 | .000 | [.33, .73] |
|  |  |  |  |  |  |  |  |  |  |  |  |  |  |  |  |
| Q3b Category |  |  |  |  |  |  |  |  |  |  |  |  |  |  |  |
| (Neither agree nor disagree) | 77 | 12 | .002 |  |  |  |  |  | 67 | 67 | .000 |  |  |  |  |
| Agree or Strongly Agree | 65 |  |  |  | .58 | .020 | [.37, .92] |  | 40 |  |  |  | .41 | .000 | [.27, .61] |
| Disagree or Strongly Disagree | 76 |  |  |  | .98 | .938 | [.60, 1.6] |  | 67 |  |  |  | .89 | .627 | [.55, 1.4] |
|  |  |  |  |  |  |  |  |  |  |  |  |  |  |  |  |
| Interaction Terms (If Significant) |  |  |  |  |  |  |  |  |  |  |  |  |  |  |  |
| Female X Humanities |  |  |  |  | 1.3 | .697 | [.33, 5.2] |  |  |  |  |  | .44 | .070 | [.18, 1.1] |
| Female X Physical Sciences |  |  |  |  | 3.2 | .069 | [.91, 11] |  |  |  |  |  | .36 | .054 | [.13, 1.0] |
| Female X Social Sciences |  |  |  |  | 2.0 | .147 | [.79, 5.0] |  |  |  |  |  | .44 | .043 | [.20, .97] |
|  |  |  |  |  |  |  |  |  |  |  |  |  |  |  |  |
| Model fit statistics |  |  |  |  |  |  |  |  |  |  |  |  |  |  |  |
| N |  |  |  |  | 738 |  |  |  |  |  |  |  | 938 |  |  |
| Pseudo R2 |  |  |  |  | .0626 |  |  |  |  |  |  |  | .1946 |  |  |
| Log likelihood |  |  |  |  | -415 |  |  |  |  |  |  |  | -523 |  |  |

Bivariate analyses for binary variables are pr-tests while non-binary variables are Chi2 tests.

| **Minimizing Harm, with an opinion (1-3 scale)** | | | | | | | | | | | | | | | | | |
| --- | --- | --- | --- | --- | --- | --- | --- | --- | --- | --- | --- | --- | --- | --- | --- | --- | --- |
|  | Students | | | | | | | |  | Faculty | | | | | | | |
|  | Bivariate Analyses | | | |  | Multivariate Analyses | | |  | Bivariate Analyses | | | |  | Multivariate Analyses | | |
| Variables | Mean | SD | Value | p-value |  | Odds Ratio | | 95% CI |  | Mean | SD | Value | p-value |  | Odds Ratio | | 95% CI |
| Respondent characteristics |  |  |  |  |  |  |  |  |  |  |  |  |  |  |  |  |  |
| All | 3.8 | 1.1 |  |  |  |  |  |  |  | 4.4 | .75 |  |  |  |  |  |  |
|  |  |  |  |  |  |  |  |  |  |  |  |  |  |  |  |  |  |
| Gender |  |  |  |  |  |  |  |  |  |  |  |  |  |  |  |  |  |
| (Male) | 4.1 | .98 | 2.2 | .025 |  |  |  |  |  | 4.4 | .70 | 1.6 | .109 |  |  |  |  |
| Female | 3.8 | 1.0 |  |  |  | .53 | .111 | [.24, 1.2] |  | 4.3 | .78 |  |  |  | .95 | .847 | [.53, 1.7] |
|  |  |  |  |  |  |  |  |  |  |  |  |  |  |  |  |  |  |
| Division |  |  |  |  |  |  |  |  |  |  |  |  |  |  |  |  |  |
| (Biological Sciences) | 4.0 | .87 | 6.6 | .086 |  |  |  |  |  | 4.6 | .61 | 65 | .000 |  |  |  |  |
| Physical Sciences | 4.0 | .91 |  |  |  | .58 | .291 | [.22, 1.6] |  | 4.3 | .89 |  |  |  | .57 | .083 | [.30, 1.1] |
| Social Sciences | 3.6 | 1.3 |  |  |  | .85 | .794 | [.25, 2.9] |  | 4.1 | .79 |  |  |  | .33 | .001 | [.17, .66] |
| Humanities | 3.1 | 1.5 |  |  |  | .13 | .031 | [.02, .83] |  | 3.8 | .76 |  |  |  | .11 | .000 | [.05, .23] |
|  |  |  |  |  |  |  |  |  |  |  |  |  |  |  |  |  |  |
| Year in School |  |  |  |  |  |  |  |  |  |  |  |  |  |  |  |  |  |
| (Freshman) | 3.6 | 1.3 | 3.5 | .322 |  |  |  |  |  |  |  |  |  |  |  |  |  |
| Sophomore | 3.8 | .89 |  |  |  | 1.3 | .489 | [.59, 3.0] |  |  |  |  |  |  |  |  |  |
| Junior | 3.9 | .97 |  |  |  | 1.3 | .490 | [.61, 2.8] |  |  |  |  |  |  |  |  |  |
| Senior | 4.0 | 1.1 |  |  |  | 1.6 | .231 | [.74, 3.5] |  |  |  |  |  |  |  |  |  |
|  |  |  |  |  |  |  |  |  |  |  |  |  |  |  |  |  |  |
| Academic Rank |  |  |  |  |  |  |  |  |  |  |  |  |  |  |  |  |  |
| (Assistant Professor) |  |  |  |  |  |  |  |  |  | 4.4 | .70 | .11 | .946 |  |  |  |  |
| Associate Professor |  |  |  |  |  |  |  |  |  | 4.3 | .95 |  |  |  | .82 | .549 | [.42, 1.6] |
| Full Professor |  |  |  |  |  |  |  |  |  | 4.4 | .70 |  |  |  | .79 | .381 | [.47, 1.3] |
|  |  |  |  |  |  |  |  |  |  |  |  |  |  |  |  |  |  |
| QIVB Category |  |  |  |  |  |  |  |  |  |  |  |  |  |  |  |  |  |
| (Neither agree nor disagree) | 3.6 | 1.1 | 36 | .000 |  |  |  |  |  | 4.2 | .70 | 55 | .000 |  |  |  |  |
| Agree or Strongly Agree | 4.2 | .79 |  |  |  | 2.1 | .056 | [.98, 4.5] |  | 4.5 | .61 |  |  |  | 2.8 | .000 | [1.6, 4.9] |
| Disagree or Strongly Disagree | 3.3 | 1.2 |  |  |  | .47 | .065 | [.21, 1.0] |  | 3.7 | 1.0 |  |  |  | .48 | .039 | [.23, .96] |
|  |  |  |  |  |  |  |  |  |  |  |  |  |  |  |  |  |  |
| Interaction Terms (If Significant) |  |  |  |  |  |  |  |  |  |  |  |  |  |  |  |  |  |
| Female X Humanities |  |  |  |  |  | 3.0 | .340 | [.31, 29] |  |  |  |  |  |  | 2.0 | .243 | [.63, 6.4] |
| Female X Physical Sciences |  |  |  |  |  | 2.2 | .494 | [.23, 20] |  |  |  |  |  |  | 2.6 | .259 | [.50, 14] |
| Female X Social Sciences |  |  |  |  |  | .80 | .771 | [.18, 3.6] |  |  |  |  |  |  | .86 | .771 | [.30, 2.5] |
|  |  |  |  |  |  |  |  |  |  |  |  |  |  |  |  |  |  |
| Model fit statistics |  |  |  |  |  |  |  |  |  |  |  |  |  |  |  |  |  |
| N |  |  |  |  |  | 213 |  |  |  |  |  |  |  |  | 465 |  |  |
| Pseudo R2 |  |  |  |  |  | .0788 |  |  |  |  |  |  |  |  | .1297 |  |  |
| Log likelihood |  |  |  |  |  | -262 |  |  |  |  |  |  |  |  | -411 |  |  |

Bivariate analyses for binary variables are Wilcoxon/Mann-Whitney tests while non-binary variables are Kruskal-Wallis tests.
