# Supplementary material for: Assessing undergraduate student and faculty views on animal research: What do they know, whom do they trust, and how much do they care?
Source: PLoS One. 2019 Oct 24;14(10):e0223375. doi: 10.1371/journal.pone.0223375 (PMC6812826; doi:10.1371/journal.pone.0223375)
Supplement: S7 Table — (DOCX) [file pone.0223375.s007.docx]

| **S7 Table. Sufficiency, % don’t know** | | | | | | | | | | | | | | | |
| --- | --- | --- | --- | --- | --- | --- | --- | --- | --- | --- | --- | --- | --- | --- | --- |
|  | Students | | | | | | |  | Faculty | | | | | | |
|  | Bivariate Analyses | | |  | Multivariate Analyses | | |  | Bivariate Analyses | | |  | Multivariate Analyses | | |
| Variables | Proportion | Value | p-value |  | Odds Ratio | | 95% CI |  | Proportion | Value | p-value |  | Odds Ratio | | 95% CI |
| Respondent characteristics |  |  |  |  |  |  |  |  |  |  |  |  |  |  |  |
| All | 66 |  |  |  |  |  |  |  | 43 |  |  |  |  |  |  |
|  |  |  |  |  |  |  |  |  |  |  |  |  |  |  |  |
| Gender |  |  |  |  |  |  |  |  |  |  |  |  |  |  |  |
| (Male) | 67 | .03 | .979 |  |  |  |  |  | 39 | -3.3 | .001 |  |  |  |  |
| Female | 67 |  |  |  | 1.2 | .481 | [.72, 2.0] |  | 51 |  |  |  | 2.5 | .003 | [1.4, 4.6] |
|  |  |  |  |  |  |  |  |  |  |  |  |  |  |  |  |
| Division |  |  |  |  |  |  |  |  |  |  |  |  |  |  |  |
| (Biological Sciences) | 59 | 17 | .001 |  |  |  |  |  | 16 | 173 | .000 |  |  |  |  |
| Physical Sciences | 76 |  |  |  | 2.5 | .004 | [1.3, 4.6] |  | 61 |  |  |  | 12 | .000 | [7.0, 19] |
| Social Sciences | 72 |  |  |  | 1.6 | .184 | [.80, 3.3] |  | 58 |  |  |  | 9.0 | .000 | [5.3, 15] |
| Humanities | 68 |  |  |  | 2.1 | .179 | [.71, 6.3] |  | 62 |  |  |  | 7.2 | .000 | [4.1, 12] |
|  |  |  |  |  |  |  |  |  |  |  |  |  |  |  |  |
| Year in School |  |  |  |  |  |  |  |  |  |  |  |  |  |  |  |
| (Freshman) | 71 | 14 | .004 |  |  |  |  |  |  |  |  |  |  |  |  |
| Sophomore | 72 |  |  |  | .94 | .797 | [.58, 1.5] |  |  |  |  |  |  |  |  |
| Junior | 64 |  |  |  | .65 | .057 | [.42, 1.0] |  |  |  |  |  |  |  |  |
| Senior | 56 |  |  |  | .47 | .001 | [.30, .74] |  |  |  |  |  |  |  |  |
|  |  |  |  |  |  |  |  |  |  |  |  |  |  |  |  |
| Academic Rank |  |  |  |  |  |  |  |  |  |  |  |  |  |  |  |
| (Assistant Professor) |  |  |  |  |  |  |  |  | 55 | 15 | .000 |  |  |  |  |
| Associate Professor |  |  |  |  |  |  |  |  | 43 |  |  |  | .58 | .025 | [.36, .94] |
| Full Professor |  |  |  |  |  |  |  |  | 39 |  |  |  | .50 | .001 | [.34, .74] |
|  |  |  |  |  |  |  |  |  |  |  |  |  |  |  |  |
| QIVB Category |  |  |  |  |  |  |  |  |  |  |  |  |  |  |  |
| (Neither agree nor disagree) | 77 | 15 | .001 |  |  |  |  |  | 63 | 73 | .000 |  |  |  |  |
| Agree or Strongly Agree | 60 |  |  |  | .45 | .001 | [.29, .72] |  | 32 |  |  |  | .34 | .000 | [.23, .50] |
| Disagree or Strongly Disagree | 67 |  |  |  | .60 | .038 | [.38, .97] |  | 57 |  |  |  | .66 | .079 | [.41, 1.1] |
|  |  |  |  |  |  |  |  |  |  |  |  |  |  |  |  |
| Interaction Terms (If Significant) |  |  |  |  |  |  |  |  |  |  |  |  |  |  |  |
| Female X Humanities |  |  |  |  | .57 | .408 | [.15, 2.2] |  |  |  |  |  | .69 | .419 | [.28, 1.7] |
| Female X Physical Sciences |  |  |  |  | 1.2 | .688 | [.43, 3.6] |  |  |  |  |  | .32 | .033 | [.11, .91] |
| Female X Social Sciences |  |  |  |  | 1.2 | .683 | [.50, 2.9] |  |  |  |  |  | .35 | .011 | [.15, .79] |
|  |  |  |  |  |  |  |  |  |  |  |  |  |  |  |  |
| Model fit statistics |  |  |  |  |  |  |  |  |  |  |  |  |  |  |  |
| N |  |  |  |  | 738 |  |  |  |  |  |  |  | 940 |  |  |
| Pseudo R2 |  |  |  |  | .0566 |  |  |  |  |  |  |  | .1964 |  |  |
| Log likelihood |  |  |  |  | -444 |  |  |  |  |  |  |  | -517 |  |  |

Bivariate analyses for binary variables are pr-tests while non-binary variables are Chi2 tests.

| **Sufficiency, with an opinion (1-3 scale)** | | | | | | | | | | | | | | | | | |
| --- | --- | --- | --- | --- | --- | --- | --- | --- | --- | --- | --- | --- | --- | --- | --- | --- | --- |
|  | Students | | | | | | | |  | Faculty | | | | | | | |
|  | Bivariate Analyses | | | |  | Multivariate Analyses | | |  | Bivariate Analyses | | | |  | Multivariate Analyses | | |
| Variables | Mean | SD | Value | p-value |  | Odds Ratio | | 95% CI |  | Mean | SD | Value | p-value |  | Odds Ratio | | 95% CI |
| Respondent characteristics |  |  |  |  |  |  |  |  |  |  |  |  |  |  |  |  |  |
| All | 2.2 | .59 |  |  |  |  |  |  |  | 2.0 | .48 |  |  |  |  |  |  |
|  |  |  |  |  |  |  |  |  |  |  |  |  |  |  |  |  |  |
| Gender |  |  |  |  |  |  |  |  |  |  |  |  |  |  |  |  |  |
| (Male) | 2.0 | .56 | -3.2 | .002 |  |  |  |  |  | 1.9 | .45 | -2.7 | .008 |  |  |  |  |
| Female | 2.2 | .57 |  |  |  | 2.3 | .046 | [1.0, 5.0] |  | 2.1 | .50 |  |  |  | 1.4 | .335 | [.70, 2.8] |
|  |  |  |  |  |  |  |  |  |  |  |  |  |  |  |  |  |  |
| Division |  |  |  |  |  |  |  |  |  |  |  |  |  |  |  |  |  |
| (Biological Sciences) | 2.2 | .48 | 4.8 | .184 |  |  |  |  |  | 1.8 | .44 | 90 | .000 |  |  |  |  |
| Physical Sciences | 2.0 | .66 |  |  |  | .55 | .237 | [.20, 1.5] |  | 2.0 | .35 |  |  |  | 3.2 | .006 | [1.4, 7.1] |
| Social Sciences | 2.2 | .75 |  |  |  | 1.6 | .435 | [.48, 5.6] |  | 2.2 | .45 |  |  |  | 8.0 | .000 | [3.2, 20] |
| Humanities | 2.2 | .71 |  |  |  | .46 | .439 | [.07, 3.3] |  | 2.4 | .49 |  |  |  | 19 | .000 | [7.3, 48] |
|  |  |  |  |  |  |  |  |  |  |  |  |  |  |  |  |  |  |
| Year in School |  |  |  |  |  |  |  |  |  |  |  |  |  |  |  |  |  |
| (Freshman) | 2.2 | .63 | 9.0 | .030 |  |  |  |  |  |  |  |  |  |  |  |  |  |
| Sophomore | 2.4 | .64 |  |  |  | 1.9 | .151 | [.79, 4.7] |  |  |  |  |  |  |  |  |  |
| Junior | 2.1 | .57 |  |  |  | .72 | .431 | [.33, 1.6] |  |  |  |  |  |  |  |  |  |
| Senior | 2.1 | .51 |  |  |  | .71 | .385 | [.32, 1.6] |  |  |  |  |  |  |  |  |  |
|  |  |  |  |  |  |  |  |  |  |  |  |  |  |  |  |  |  |
| Academic Rank |  |  |  |  |  |  |  |  |  |  |  |  |  |  |  |  |  |
| (Assistant Professor) |  |  |  |  |  |  |  |  |  | 2.0 | .45 | .86 | .652 |  |  |  |  |
| Associate Professor |  |  |  |  |  |  |  |  |  | 2.0 | .57 |  |  |  | .97 | .927 | [.47, 2.0] |
| Full Professor |  |  |  |  |  |  |  |  |  | 2.0 | .46 |  |  |  | .85 | .603 | [.47, 1.6] |
|  |  |  |  |  |  |  |  |  |  |  |  |  |  |  |  |  |  |
| QIVB Category |  |  |  |  |  |  |  |  |  |  |  |  |  |  |  |  |  |
| (Neither agree nor disagree) | 2.1 | .41 | 44 | .000 |  |  |  |  |  | 2.1 | .48 | 86 | .000 |  |  |  |  |
| Agree or Strongly Agree | 2.0 | .43 |  |  |  | .60 | .242 | [.26, 1.4] |  | 1.9 | .39 |  |  |  | .29 | .002 | [.13, .63] |
| Disagree or Strongly Disagree | 2.5 | .72 |  |  |  | 4.9 | .000 | [2.0, 12] |  | 2.4 | .58 |  |  |  | 4.1 | .001 | [1.7, 9.7] |
|  |  |  |  |  |  |  |  |  |  |  |  |  |  |  |  |  |  |
| Interaction Terms (If Significant) |  |  |  |  |  |  |  |  |  |  |  |  |  |  |  |  |  |
| Female X Humanities |  |  |  |  |  | 2.6 | .434 | [.24, 27] |  |  |  |  |  |  | .84 | .811 | [.20, 3.5] |
| Female X Physical Sciences |  |  |  |  |  | .49 | .489 | [.07, 3.7] |  |  |  |  |  |  | .82 | .843 | [.11, 6.0] |
| Female X Social Sciences |  |  |  |  |  | .31 | .136 | [.07, 1.4] |  |  |  |  |  |  | .92 | .898 | [.26, 3.3] |
|  |  |  |  |  |  |  |  |  |  |  |  |  |  |  |  |  |  |
| Model fit statistics |  |  |  |  |  |  |  |  |  |  |  |  |  |  |  |  |  |
| N |  |  |  |  |  | 247 |  |  |  |  |  |  |  |  | 533 |  |  |
| Pseudo R2 |  |  |  |  |  | .1498 |  |  |  |  |  |  |  |  | .2325 |  |  |
| Log likelihood |  |  |  |  |  | -185 |  |  |  |  |  |  |  |  | -285 |  |  |

Bivariate analyses for binary variables are Wilcoxon/Mann-Whitney tests while non-binary variables are Kruskal-Wallis tests.
