# Supplementary material for: Assessing undergraduate student and faculty views on animal research: What do they know, whom do they trust, and how much do they care?
Source: PLoS One. 2019 Oct 24;14(10):e0223375. doi: 10.1371/journal.pone.0223375 (PMC6812826; doi:10.1371/journal.pone.0223375)
Supplement: S8 Table — (DOCX) [file pone.0223375.s008.docx]

| **S8 Table. Informed Decisions, percent don’t know: Bivariate and Multivariate Analyses** | | | | | | | | | | | | | | | |
| --- | --- | --- | --- | --- | --- | --- | --- | --- | --- | --- | --- | --- | --- | --- | --- |
|  | Students | | | | | | |  | Faculty | | | | | | |
|  | Bivariate Analyses | | |  | Multivariate Analyses | | |  | Bivariate Analyses | | |  | Multivariate Analyses | | |
| Variables | Proportion | Value | p-value |  | Odds Ratio | | 95% CI |  | Proportion | Value | p-value |  | Odds Ratio | | 95% CI |
| Respondent characteristics |  |  |  |  |  |  |  |  |  |  |  |  |  |  |  |
| All | 12 |  |  |  |  |  |  |  | 7 |  |  |  |  |  |  |
|  |  |  |  |  |  |  |  |  |  |  |  |  |  |  |  |
| Gender |  |  |  |  |  |  |  |  |  |  |  |  |  |  |  |
| (Male) | 16 | 2.3 | .025 |  |  |  |  |  | 7 | -1.2 | .221 |  |  |  |  |
| Female | 10 |  |  |  | .45 | .045 | [.21, .98] |  | 9 |  |  |  | 3.0 | .130 | [.73, 12] |
|  |  |  |  |  |  |  |  |  |  |  |  |  |  |  |  |
| Division |  |  |  |  |  |  |  |  |  |  |  |  |  |  |  |
| (Biological Sciences) | 9 | 3.1 | .370 |  |  |  |  |  | 2 | 24 | .000 |  |  |  |  |
| Physical Sciences | 14 |  |  |  | 1.1 | .781 | [.51, 2.5] |  | 12 |  |  |  | 8.2 | .000 | [2.7, 25] |
| Social Sciences | 14 |  |  |  | .72 | .528 | [.26, 2.0] |  | 9 |  |  |  | 6.2 | .002 | [1.9, 20] |
| Humanities | 13 |  |  |  | .92 | .910 | [.23, 3.7] |  | 11 |  |  |  | 8.5 | .000 | [2.6, 28] |
|  |  |  |  |  |  |  |  |  |  |  |  |  |  |  |  |
| Year in School |  |  |  |  |  |  |  |  |  |  |  |  |  |  |  |
| (Freshman) | 14 | 4.1 | .248 |  |  |  |  |  |  |  |  |  |  |  |  |
| Sophomore | 15 |  |  |  | 1.1 | .776 | [.59, 2.0] |  |  |  |  |  |  |  |  |
| Junior | 11 |  |  |  | .73 | .334 | [.39, 1.4] |  |  |  |  |  |  |  |  |
| Senior | 9 |  |  |  | .58 | .107 | [.30, 1.1] |  |  |  |  |  |  |  |  |
|  |  |  |  |  |  |  |  |  |  |  |  |  |  |  |  |
| Academic Rank |  |  |  |  |  |  |  |  |  |  |  |  |  |  |  |
| (Assistant Professor) |  |  |  |  |  |  |  |  | 9 | .65 | .724 |  |  |  |  |
| Associate Professor |  |  |  |  |  |  |  |  | 7 |  |  |  | .81 | .600 | [.38, 1.8] |
| Full Professor |  |  |  |  |  |  |  |  | 7 |  |  |  | .82 | .521 | [.44, 1.5] |
|  |  |  |  |  |  |  |  |  |  |  |  |  |  |  |  |
| QIVB Category |  |  |  |  |  |  |  |  |  |  |  |  |  |  |  |
| (Neither agree nor disagree) | 11 | .75 | .687 |  |  |  |  |  | 12 | 6.9 | .032 |  |  |  |  |
| Agree or Strongly Agree | 12 |  |  |  | 1.1 | .826 | [.56, 2.1] |  | 7 |  |  |  | .67 | .183 | [.38, 1.2] |
| Disagree or Strongly Disagree | 14 |  |  |  | 1.4 | .256 | [.76, 2.7] |  | 6 |  |  |  | .38 | .016 | [.18, .83] |
|  |  |  |  |  |  |  |  |  |  |  |  |  |  |  |  |
| Interaction Terms (If Significant) |  |  |  |  |  |  |  |  |  |  |  |  |  |  |  |
| Female X Humanities |  |  |  |  | 1.6 | .596 | [.27, 9.7] |  |  |  |  |  | .37 | .258 | [.07, 2.1] |
| Female X Physical Sciences |  |  |  |  | .81 | .777 | [.18, 3.6] |  |  |  |  |  | .51 | .475 | [.08, 3.2] |
| Female X Social Sciences |  |  |  |  | 2.9 | .088 | [.85, 9.9] |  |  |  |  |  | .40 | .284 | [.08, 2.1] |
|  |  |  |  |  |  |  |  |  |  |  |  |  |  |  |  |
| Model fit statistics |  |  |  |  |  |  |  |  |  |  |  |  |  |  |  |
| N |  |  |  |  | 737 |  |  |  |  |  |  |  | 935 |  |  |
| Pseudo R2 |  |  |  |  | .0286 |  |  |  |  |  |  |  | .0748 |  |  |
| Log likelihood |  |  |  |  | -257 |  |  |  |  |  |  |  | -230 |  |  |

Bivariate analyses for binary variables are pr-tests while non-binary variables are Chi2 tests.

| **Informed Decisions, with an opinion (1-5 Scale)** | | | | | | | | | | | | | | | | | |
| --- | --- | --- | --- | --- | --- | --- | --- | --- | --- | --- | --- | --- | --- | --- | --- | --- | --- |
|  | Students | | | | | | | |  | Faculty | | | | | | | |
|  | Bivariate Analyses | | | |  | Multivariate Analyses | | |  | Bivariate Analyses | | | |  | Multivariate Analyses | | |
| Variables | Mean | SD | Value | p-value |  | Odds Ratio | | 95% CI |  | Mean | SD | Value | p-value |  | Odds Ratio | | 95% CI |
| Respondent characteristics |  |  |  |  |  |  |  |  |  |  |  |  |  |  |  |  |  |
| All | 2.8 | 1.2 |  |  |  |  |  |  |  | 3.4 | 1.2 |  |  |  |  |  |  |
|  |  |  |  |  |  |  |  |  |  |  |  |  |  |  |  |  |  |
| Gender |  |  |  |  |  |  |  |  |  |  |  |  |  |  |  |  |  |
| (Male) | 3.0 | 1.1 | 2.9 | .004 |  |  |  |  |  | 3.5 | 1.2 | 4.1 | .000 |  |  |  |  |
| Female | 2.7 | 1.2 |  |  |  | .58 | .031 | [.36, .95] |  | 3.1 | 1.2 |  |  |  | .57 | .017 | [.36, .90] |
|  |  |  |  |  |  |  |  |  |  |  |  |  |  |  |  |  |  |
| Division |  |  |  |  |  |  |  |  |  |  |  |  |  |  |  |  |  |
| (Biological Sciences) | 3.0 | 1.2 | 21 | .000 |  |  |  |  |  | 4.1 | 1.0 | 233 | .000 |  |  |  |  |
| Physical Sciences | 2.7 | 1.1 |  |  |  | .47 | .008 | [.27, .83] |  | 3.0 | 1.2 |  |  |  | .12 | .000 | [.08, .18] |
| Social Sciences | 2.5 | 1.2 |  |  |  | .37 | .003 | [.20, .72] |  | 2.9 | 1.2 |  |  |  | .14 | .000 | [.09, .21] |
| Humanities | 2.7 | 1.2 |  |  |  | .77 | .604 | [.29, 2.1] |  | 2.7 | 1.0 |  |  |  | .10 | .000 | [.07, .17] |
|  |  |  |  |  |  |  |  |  |  |  |  |  |  |  |  |  |  |
| Year in School |  |  |  |  |  |  |  |  |  |  |  |  |  |  |  |  |  |
| (Freshman) | 2.7 | 1.1 | 9.7 | .021 |  |  |  |  |  |  |  |  |  |  |  |  |  |
| Sophomore | 2.6 | 1.2 |  |  |  | .74 | .146 | [.74, 1.1] |  |  |  |  |  |  |  |  |  |
| Junior | 2.9 | 1.2 |  |  |  | 1.2 | .388 | [.81, 1.7] |  |  |  |  |  |  |  |  |  |
| Senior | 3.0 | 1.2 |  |  |  | 1.4 | .063 | [.98, 2.1] |  |  |  |  |  |  |  |  |  |
|  |  |  |  |  |  |  |  |  |  |  |  |  |  |  |  |  |  |
| Academic Rank |  |  |  |  |  |  |  |  |  |  |  |  |  |  |  |  |  |
| (Assistant Professor) |  |  |  |  |  |  |  |  |  | 3.1 | 1.3 | 11 | .003 |  |  |  |  |
| Associate Professor |  |  |  |  |  |  |  |  |  | 3.4 | 1.2 |  |  |  | 1.4 | .109 | [.93, 2.0] |
| Full Professor |  |  |  |  |  |  |  |  |  | 3.5 | 1.2 |  |  |  | 1.5 | .015 | [1.1, 2.1] |
|  |  |  |  |  |  |  |  |  |  |  |  |  |  |  |  |  |  |
| QIVB Category |  |  |  |  |  |  |  |  |  |  |  |  |  |  |  |  |  |
| (Neither agree nor disagree) | 2.4 | 1.0 | 45 | .000 |  |  |  |  |  | 2.8 | 1.2 | 94 | .000 |  |  |  |  |
| Agree or Strongly Agree | 3.1 | 1.2 |  |  |  | 3.0 | .000 | [2.0, 4.4] |  | 3.7 | 1.2 |  |  |  | 2.9 | .000 | [2.0, 4.0] |
| Disagree or Strongly Disagree | 2.7 | 1.1 |  |  |  | 1.7 | .011 | [1.1, 2.4] |  | 2.9 | 1.2 |  |  |  | 1.5 | .036 | [1.0, 2.3] |
|  |  |  |  |  |  |  |  |  |  |  |  |  |  |  |  |  |  |
| Interaction Terms (If Significant) |  |  |  |  |  |  |  |  |  |  |  |  |  |  |  |  |  |
| Female X Humanities |  |  |  |  |  | .67 | .500 | [.20, 2.2] |  |  |  |  |  |  | 1.4 | .366 | [.67, 2.9] |
| Female X Physical Sciences |  |  |  |  |  | .90 | .807 | [.40, 2.0] |  |  |  |  |  |  | 1.6 | .348 | [.60, 4.2] |
| Female X Social Sciences |  |  |  |  |  | 1.2 | .585 | [.57, 2.7] |  |  |  |  |  |  | 1.5 | .237 | [.77, 2.9] |
|  |  |  |  |  |  |  |  |  |  |  |  |  |  |  |  |  |  |
| Model fit statistics |  |  |  |  |  |  |  |  |  |  |  |  |  |  |  |  |  |
| N |  |  |  |  |  | 651 |  |  |  |  |  |  |  |  | 865 |  |  |
| Pseudo R2 |  |  |  |  |  | .0457 |  |  |  |  |  |  |  |  | .1194 |  |  |
| Log likelihood |  |  |  |  |  | -944 |  |  |  |  |  |  |  |  | -1173 |  |  |

Bivariate analyses for binary variables are Wilcoxon/Mann-Whitney tests while non-binary variables are Kruskal-Wallis tests.
