# Supplementary material for: Assessing undergraduate student and faculty views on animal research: What do they know, whom do they trust, and how much do they care?
Source: PLoS One. 2019 Oct 24;14(10):e0223375. doi: 10.1371/journal.pone.0223375 (PMC6812826; doi:10.1371/journal.pone.0223375)
Supplement: S5 Table — (DOCX) [file pone.0223375.s005.docx]

| **S5 Table. Review, % don’t know** | | | | | | | | | | | | | | | |
| --- | --- | --- | --- | --- | --- | --- | --- | --- | --- | --- | --- | --- | --- | --- | --- |
|  | Students | | | | | | |  | Faculty | | | | | | |
|  | Bivariate Analyses | | |  | Multivariate Analyses | | |  | Bivariate Analyses | | |  | Multivariate Analyses | | |
| Variables | Proportion | Value | p-value |  | Odds Ratio | | 95% CI |  | Proportion | Value | p-value |  | Odds Ratio | | 95% CI |
| Respondent characteristics |  |  |  |  |  |  |  |  |  |  |  |  |  |  |  |
| All | 75 |  |  |  |  |  |  |  | 42 |  |  |  |  |  |  |
|  |  |  |  |  |  |  |  |  |  |  |  |  |  |  |  |
| Gender |  |  |  |  |  |  |  |  |  |  |  |  |  |  |  |
| (Male) | 77 | 1.1 | .278 |  |  |  |  |  | 38 | -3.0 | .003 |  |  |  |  |
| Female | 74 |  |  |  | .77 | .351 | [.44, 1.3] |  | 48 |  |  |  | 1.7 | .088 | [.92, 3.3] |
|  |  |  |  |  |  |  |  |  |  |  |  |  |  |  |  |
| Division |  |  |  |  |  |  |  |  |  |  |  |  |  |  |  |
| (Biological Sciences) | 66 | 30 | .000 |  |  |  |  |  | 15 | 168 | .000 |  |  |  |  |
| Physical Sciences | 84 |  |  |  | 2.8 | .004 | [1.4, 5.6] |  | 56 |  |  |  | 9.2 | .000 | [5.6, 15] |
| Social Sciences | 84 |  |  |  | 1.6 | .222 | [.74, 3.6] |  | 57 |  |  |  | 7.9 | .000 | [4.6, 13] |
| Humanities | 77 |  |  |  | 2.1 | .244 | [.60, 7.4] |  | 61 |  |  |  | 8.5 | .000 | [4.8, 15] |
|  |  |  |  |  |  |  |  |  |  |  |  |  |  |  |  |
| Year in School |  |  |  |  |  |  |  |  |  |  |  |  |  |  |  |
| (Freshman) | 83 | 22 | .000 |  |  |  |  |  |  |  |  |  |  |  |  |
| Sophomore | 78 |  |  |  | .83 | .515 | [.48, 1.4] |  |  |  |  |  |  |  |  |
| Junior | 72 |  |  |  | .54 | .017 | [.33, .90] |  |  |  |  |  |  |  |  |
| Senior | 63 |  |  |  | .33 | .000 | [.20, .55] |  |  |  |  |  |  |  |  |
|  |  |  |  |  |  |  |  |  |  |  |  |  |  |  |  |
| Academic Rank |  |  |  |  |  |  |  |  |  |  |  |  |  |  |  |
| (Assistant Professor) |  |  |  |  |  |  |  |  | 54 | 20 | .000 |  |  |  |  |
| Associate Professor |  |  |  |  |  |  |  |  | 45 |  |  |  | .77 | .274 | [.48, 1.2] |
| Full Professor |  |  |  |  |  |  |  |  | 36 |  |  |  | .48 | .000 | [.33, .70] |
|  |  |  |  |  |  |  |  |  |  |  |  |  |  |  |  |
| QIVB Category |  |  |  |  |  |  |  |  |  |  |  |  |  |  |  |
| (Neither agree nor disagree) | 82 | 25 | .000 |  |  |  |  |  | 54 | 49 | .000 |  |  |  |  |
| Agree or Strongly Agree | 66 |  |  |  | .42 | .001 | [.26, .70] |  | 33 |  |  |  | .57 | .004 | [.39, .84] |
| Disagree or Strongly Disagree | 81 |  |  |  | .99 | .974 | [.58, 1.7] |  | 57 |  |  |  | 1.0 | .879 | [.66, 1.6] |
|  |  |  |  |  |  |  |  |  |  |  |  |  |  |  |  |
| Interaction Terms (If Significant) |  |  |  |  |  |  |  |  |  |  |  |  |  |  |  |
| Female X Humanities |  |  |  |  | .65 | .578 | [.15, 2.9] |  |  |  |  |  | .62 | .295 | [.25, 1.5] |
| Female X Physical Sciences |  |  |  |  | 1.1 | .898 | [.34, 3.4] |  |  |  |  |  | .57 | .297 | [.20, 1.6] |
| Female X Social Sciences |  |  |  |  | 1.9 | .197 | [.71, 5.3] |  |  |  |  |  | .61 | .239 | [.27, 1.4] |
|  |  |  |  |  |  |  |  |  |  |  |  |  |  |  |  |
| Model fit statistics |  |  |  |  |  |  |  |  |  |  |  |  |  |  |  |
| N |  |  |  |  | 738 |  |  |  |  |  |  |  | 939 |  |  |
| Pseudo R2 |  |  |  |  | .0994 |  |  |  |  |  |  |  | .1774 |  |  |
| Log likelihood |  |  |  |  | -376 |  |  |  |  |  |  |  | -525 |  |  |

Bivariate analyses for binary variables are pr-tests while non-binary variables are Chi2 tests.

| **Review, with an opinion (1-5 scale)** | | | | | | | | | | | | | | | | | |
| --- | --- | --- | --- | --- | --- | --- | --- | --- | --- | --- | --- | --- | --- | --- | --- | --- | --- |
|  | Students | | | | | | | |  | Faculty | | | | | | | |
|  | Bivariate Analyses | | | |  | Multivariate Analyses | | |  | Bivariate Analyses | | | |  | Multivariate Analyses | | |
| Variables | Mean | SD | Value | p-value |  | Odds Ratio | | 95% CI |  | Mean | SD | Value | p-value |  | Odds Ratio | | 95% CI |
| Respondent characteristics |  |  |  |  |  |  |  |  |  |  |  |  |  |  |  |  |  |
| All | 3.9 | .98 |  |  |  |  |  |  |  | 4.4 | .65 |  |  |  |  |  |  |
|  |  |  |  |  |  |  |  |  |  |  |  |  |  |  |  |  |  |
| Gender |  |  |  |  |  |  |  |  |  |  |  |  |  |  |  |  |  |
| (Male) | 3.9 | .90 | .31 | .754 |  |  |  |  |  | 4.4 | .64 | .29 | .775 |  |  |  |  |
| Female | 3.9 | .93 |  |  |  | 1.1 | .781 | [.50, 2.5] |  | 4.4 | .62 |  |  |  | .83 | .506 | [.49, 1.4] |
|  |  |  |  |  |  |  |  |  |  |  |  |  |  |  |  |  |  |
| Division |  |  |  |  |  |  |  |  |  |  |  |  |  |  |  |  |  |
| (Biological Sciences) | 4.1 | .79 | 15 | .002 |  |  |  |  |  | 4.5 | .59 | 40 | .000 |  |  |  |  |
| Physical Sciences | 3.8 | .94 |  |  |  | .98 | .973 | [.30, 3.2] |  | 4.2 | .75 |  |  |  | .35 | .000 | [.20, .60] |
| Social Sciences | 3.5 | 1.0 |  |  |  | .52 | .309 | [.15, 1.8] |  | 4.4 | .56 |  |  |  | .61 | .107 | [.34, 1.1] |
| Humanities | 3.1 | 1.4 |  |  |  | .09 | .057 | [.01, 1.1] |  | 4.0 | .73 |  |  |  | .19 | .000 | [.09, .39] |
|  |  |  |  |  |  |  |  |  |  |  |  |  |  |  |  |  |  |
| Year in School |  |  |  |  |  |  |  |  |  |  |  |  |  |  |  |  |  |
| (Freshman) | 3.5 | 1.1 | 10 | .015 |  |  |  |  |  |  |  |  |  |  |  |  |  |
| Sophomore | 3.7 | .91 |  |  |  | 1.1 | .917 | [.41, 2.7] |  |  |  |  |  |  |  |  |  |
| Junior | 3.9 | .90 |  |  |  | 1.5 | .393 | [.61, 3.6] |  |  |  |  |  |  |  |  |  |
| Senior | 4.1 | .93 |  |  |  | 2.7 | .026 | [1.1, 6.4] |  |  |  |  |  |  |  |  |  |
|  |  |  |  |  |  |  |  |  |  |  |  |  |  |  |  |  |  |
| Academic Rank |  |  |  |  |  |  |  |  |  |  |  |  |  |  |  |  |  |
| (Assistant Professor) |  |  |  |  |  |  |  |  |  | 4.5 | .66 | 1.1 | .584 |  |  |  |  |
| Associate Professor |  |  |  |  |  |  |  |  |  | 4.3 | .77 |  |  |  | .61 | .103 | [.33, 1.1] |
| Full Professor |  |  |  |  |  |  |  |  |  | 4.4 | .61 |  |  |  | .81 | .406 | [.50, 1.3] |
|  |  |  |  |  |  |  |  |  |  |  |  |  |  |  |  |  |  |
| QIVB Category |  |  |  |  |  |  |  |  |  |  |  |  |  |  |  |  |  |
| (Neither agree nor disagree) | 3.6 | .99 | 27 | .000 |  |  |  |  |  | 4.2 | .71 | 41 | .000 |  |  |  |  |
| Agree or Strongly Agree | 4.2 | .80 |  |  |  | 2.5 | .043 | [1.0, 5.9] |  | 4.5 | .54 |  |  |  | 2.8 | .000 | [1.7, 4.5] |
| Disagree or Strongly Disagree | 3.3 | 1.1 |  |  |  | .67 | .407 | [.26, 1.7] |  | 4.1 | .83 |  |  |  | .69 | .259 | [.36, 1.3] |
|  |  |  |  |  |  |  |  |  |  |  |  |  |  |  |  |  |  |
| Interaction Terms (If Significant) |  |  |  |  |  |  |  |  |  |  |  |  |  |  |  |  |  |
| Female X Humanities |  |  |  |  |  | 2.9 | .466 | [.17, 49] |  |  |  |  |  |  | 1.6 | .440 | [.50, 5.0] |
| Female X Physical Sciences |  |  |  |  |  | .40 | .356 | [.06, 2.8] |  |  |  |  |  |  | 4.7 | .030 | [1.2, 19] |
| Female X Social Sciences |  |  |  |  |  | .59 | .517 | [.12, 3.0] |  |  |  |  |  |  | 2.0 | .176 | [.74, 5.2] |
|  |  |  |  |  |  |  |  |  |  |  |  |  |  |  |  |  |  |
| Model fit statistics |  |  |  |  |  |  |  |  |  |  |  |  |  |  |  |  |  |
| N |  |  |  |  |  | 187 |  |  |  |  |  |  |  |  | 548 |  |  |
| Pseudo R2 |  |  |  |  |  | .1005 |  |  |  |  |  |  |  |  | .0880 |  |  |
| Log likelihood |  |  |  |  |  | -212 |  |  |  |  |  |  |  |  | -452 |  |  |

Bivariate analyses for binary variables are Wilcoxon/Mann-Whitney tests while non-binary variables are Kruskal-Wallis tests.
